#  Australian gall-inducing scale insects on *Eucalyptus*: revision of *Opisthoscelis* Schrader (Coccoidea, Eriococcidae) and descriptions of a new genus and nine new species

**DOI:** 10.3897/zookeys.58.507

**Published:** 2010-09-24

**Authors:** Nate B. Hardy, Penny J. Gullan

**Affiliations:** 1 Entomology, Queensland Department of Employment, Economic Development and Innovation, 80 Meiers Road, Indooroopilly, Queensland 4068, Australia; 2 Department of Entomology, University of California, One Shields Avenue, Davis, California 95616-8584, USA

**Keywords:** taxonomy, systematics, felt scales, galls

## Abstract

We revise the genus Opisthoscelis Schrader, and erect the genus Tanyscelis **gen. n.** with Opisthoscelis pisiformis Froggatt as its type species. Species of both genera induce sexually dimorphic galls on Eucalyptus (Myrtaceae) in Australia, with Opisthoscelis subrotunda Schrader also in Papua New Guinea. We synonymise the following taxa (junior synonym with senior synonym): Opisthoscelis fibularis Froggatt, **syn. n.** with Opisthoscelis spinosa Froggatt; Opisthoscelis recurva Froggatt, **syn. n.** with Opisthoscelis maculata Froggatt; Opisthoscelis globosa Froggatt, **syn. n.** (= Opisthoscelis ruebsaameni Lindinger) with Opisthoscelis convexa Froggatt; and Opisthoscelis mammularis Froggatt, **syn. n.** with Opisthoscelis verrucula Froggatt. We transfer seven Opisthoscelis species to Tanyscelis as Tanyscelis conica (Fuller), **comb. n.**, Tanyscelis convexa (Froggatt), **comb. n.**, Tanyscelis maculata (Froggatt), **comb. n.**, Tanyscelis maskelli (Froggatt), **comb. n.**, Tanyscelis pisiformis (Froggatt), **comb. n.**, Tanyscelis spinosa (Froggatt), **comb. n.**, and Tanyscelis verrucula (Froggatt), **comb. n.** We redescribe and illustrate the adult female of each named species of Opisthoscelis for which the type material is known, as well as the first-instar nymph of the type species of Opisthoscelis (Opisthoscelis subrotunda) and Tanyscelis (Opisthoscelis pisiformis). We describe four new species of Opisthoscelis: Opisthoscelis beardsleyi Hardy & Gullan, **sp. n.**, Opisthoscelis thurgoona Hardy & Gullan, **sp. n.**, Opisthoscelis tuberculataHardy & Gullan, **sp. n.**, and Opisthoscelis ungulifinis Hardy & Gullan, **sp. n.**, and five new species of Tanyscelis: Tanyscelis grallator Hardy & Gullan, **sp. n.**, Tanuscelis megagibba Hardy & Gullan, **sp. n.**, Tanyscelis mollicornuta Hardy & Gullan, **sp. n.**, Tanyscelis tripocula Hardy & Gullan, **sp. n.**, and Tanyscelis villosigibba Hardy & Gullan, **sp. n.** We designate lectotypes for Opisthoscelis convexa, Opisthoscelis fibularis, Opisthoscelis globosa Froggatt, Opisthoscelis maculata, Opisthoscelis mammularis, Opisthoscelis maskelli, Opisthoscelis pisiformis, Opisthoscelis recurva, Opisthoscelis serrata, Opisthoscelis spinosa, and Opisthoscelis verrucula. As a result of our taxonomic revision, Opisthoscelis has six species and Tanyscelis has 12 species. We describe the galls of females for all 18 species and galls of males for 10 species of Opisthoscelis and Tanyscelis, and provide photographs of the galls for most species. A key to the adult females of the species of both genera is included.

## Introduction

Price (2005) pointed out that gall-inducing insect taxa tend to be species-rich when they specialise on species-rich host groups. Australian gall-inducing scale insects are no exception. A large proportion of the Australian gall-inducing scale insect fauna is associated with the hyper-diverse genus Eucalyptus (Gullan et al. 2005). Scale insect galls on Eucalyptus sensu lato are a conspicuous element of the Australian fauna (at least to botanists) and have even been incorporated into human diets (Froggatt 1893; Latz 1995). Gall-induction has evolved repeatedly in scale insects (Gullan et al. 2005), including within the Eriococcidae (Cook and Gullan 2004), the scale insect family with the most gall-inducing species. The two most species-rich radiations of gall-inducing eriococcids are parasites of Eucalyptus: (1) the genus Apiomorpha Rübsaamen (ca 40 described species and many undescribed cryptic species) (Gullan 1984a; Cook and Rowell 2007), and (2) a monophyletic group (NBH unpublished data) composed of species currently placed in the genera Lachnodius Maskell, Opisthoscelis Schrader, Sphaerococcopsis Cockerell, Fragorbis Hardy and Gullanр and Floracoccus Beardsley (ca 30 described species).

Beardsley (1974a, b) described or redescribed the four species of Sphaerococcopsis and the single species of Floracoccus. Hardy and Gullan (2007) erected the genus Fragorbis for five species that mostly induce blister galls on stems or live under bark. Taxonomic work on a number of species closely related to Lachnodius eucalypti Maskell, initiated by the late J.W. Beardsley, is currently in preparation (NHB, PJG and J.W. Beardsley unpublished data).

Schrader (1863) erected the genus Opisthoscelis for Opisthoscelis subrotunda, although Opisthoscelis subrotunda was not officially designated as the type of the genus until Fernald’s (1903) catalogue. Rübsaamen (1894) described Opisthoscelis globosa, the type material of which appears to have been destroyed in WWII (Gullan 1984a). Froggatt (1894a,b, 1898, 1929), Fuller (1897, 1899) and Kieffer and Jorgensen (1910) described 14 additional species. In his catalogue of the scale insects of Australia, Froggatt (1921) republished his earlier descriptions and illustrations of eight Opisthoscelis species and provided notes on three species described by other authors. Opisthoscelis prosopidis Kireffer and Jorgensen was described from Prosopis adesmiodes (Fabaceae) in Argentina. It is almost certainly not congeneric with Eucalyptus-feeding species in Australia (Miller and Gimpel 2000) and we do not deal with it in this paper. Unfortunately, the whereabouts of the type material of Opisthoscelis prosopidis is unknown (Miller and Gimpel 2000; Hodgson and Miller 2010). Lindinger (1943) chose Opisthoscelis ruebsaamani as a replacement name for Opisthoscelis globosa Froggatt (1929), which is a junior primary homonym of Opisthoscelis globosa Rübsaamen. Hardy and Gullan (2009) transferred Opisthoscelis nigra Froggatt, described from galls on Eucalyptus in New South Wales (Froggatt 1898), to the armoured scale genus Maskellia Fuller (Diaspididae).

Here we revise the genus Opisthoscelis, and transfer a number of Opisthoscelis species into the new genus Tanyscelis. We describe and illustrate: (1) the adult female of each of the previously named species of Opisthoscelis for which the type material is known; (2) the first-instar nymph of the type species of Opisthoscelis and Tanyscelis; and (3) the adult female for each of eight new species. We synonymise a number of species described by Froggatt. We describe the galls of females and, if available, of males of the 18 species of Opisthoscelis and Tanyscelis, and provide a key to the adult females of Opisthoscelis and Tanyscelis.

## Methods

Freshly collected specimens were slide-mounted in Canada balsam using the method described in Gullan (1984a). Adult females were mounted one specimen per slide; nymphs were mounted several to a slide. Froggatt prepared very few slide-mounts and thus his dry type galls were crucial to confirming the identity of his species. For most of the named species, adult females were dissected from dry type galls in museum collections and mounted on microscope slides by PJG. The dry insects were soaked in ®Decon 90 or ®Contrad 70 detergent for 24 hours and then placed in water to rehydrate the specimen prior to clearing of body contents in 10% KOH and then slide-mounting according to the usual method. Descriptions of all life stages are based on slide-mounted specimens. Measurements of insects were made using an ocular micrometer attached to a compound microscope. All measurements are maximum dimensions (e.g., body width was recorded at the widest point and leg segment lengths were measured along the longest axis), and are expressed as the range. Tarsal length excludes the claw. Spiracle length includes the muscle plate (apodeme). Setal lengths exclude the setal base. The length of the macrotubular ducts excludes the filament. The hind leg of the adult female is described as having a functional articulation between the femur and tibia if intersegmental membrane is present and the leg is capable of flexing at the joint. The number of specimens measured is reported in the heading for each description, although usually many more specimens were examined, as reported in the lists of ‘Material examined’. Samples in each ‘Material examined’ section were sorted in ascending order algorithmically, starting with the first character in each locality, with numeric characters having a higher priority than alphabetic characters. For example ‘11 km N of Bundoora’ would appear before ‘Atherton,’ and ‘45 km S of Brisbane’ would appear before ‘9 km E of Townsville.’

**Figure 1.**
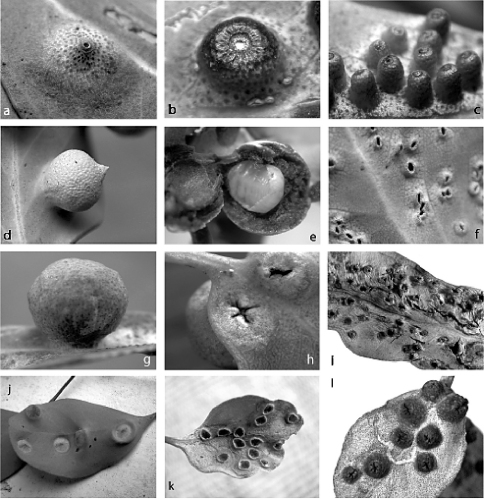
Galls of species of Opisthoscelis: Opisthoscelis beardsleyi sp. n.: **a** gall of female, ex Eucalyptus goniocalyx, Victoria, Belgrave South **b** gall of female, ex Eucalyptus viminalis, Victoria, Cranbourne **c** immature galls of males, ex Eucalyptus viminalis, Cranbourne. Opisthoscelis serrata, ex Eucalyptus polyanthemos, Victoria, Melbourne **d** gall of female **e** gall cut open to reveal adult female **f** galls of males. Opisthoscelis subrotunda: **g** gall of female, ex Eucalyptus ?camaldulensis, Victoria, Shepparton **h** galls of females showing orifice, ex Eucalyptus camaldulensis, Victoria, Grampians National Park **i** galls of males, ex Eucalyptus sp., New South Wales. Opisthoscelis thurgoona sp. n.: **j** young galls of females (four with slit-like orifice visible, top left gall showing underside), ex Eucalyptus melliodora, New South Wales, Thurgoona. Opisthoscelis tuberculatasp. n.: **k** vacated pit galls of females, ex Eucalyptus sp., Victoria, Benalla. Opisthoscelis ungulifinis sp. n.: **l** dry galls of females showing star-like orifice, ex Eucalyptus ?oleosa, South Australia, Oodla Wirra. [Photographs by PJG except i by L.A. Mound]

**Figure 2.**
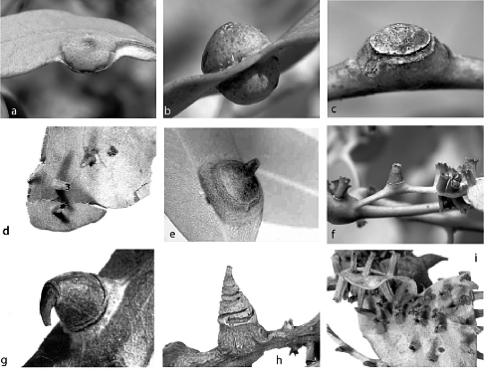
Galls of species of Tanyscelis: Tanyscelis conica, ex Eucalyptus dumosa, Victoria, Mittyack: **a** gall of female **b** gall of female, showing both sides of leaf. Tanyscelis convexa: **c** gall of female, ex Eucalyptus polyanthemos, Victoria, Melbourne **d** dry galls of males, ex Eucalyptus goniocalyx, Victoria, Dandenong. Tanyscelis grallatorsp. n.: **e** gall of female, ex ironbark, Queensland, Dunmore State Forest. Tanyscelis maculata: **f** gall of female (center of photo) and galls of males, ex Eucalyptus melliodora, Victoria, Nagambie **g** dry gall of female from type material of Opisthoscelis recurva, ex Eucalyptus sp., New South Wales, Warrah. Tanyscelis maskelli, ex Eucalyptus sp., New South Wales, Flemington, W.W. Froggatt No. 1944E: **h** dry gall of female **i** dry galls of males. [Photographs by PJG]

**Figure 3.**
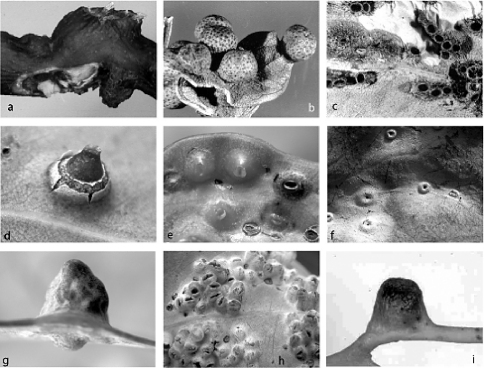
Galls of species of Tanyscelis: Tanyscelis megagibba sp. n.: **a** gall of adult female (upper), plus gall opened to show adult female (lower), ex Eucalyptus microcarpa, South Australia, Aldinga Beach. Tanyscelis pisiformis, ex Eucalyptus sp., probably from New South Wales: **b** dry galls of females **c** dry galls of males. Tanyscelis spinosa: **d** mature female gall, ex Eucalyptus ?goniocalyx sapling, Victoria, Bendigo **e** young galls of females and one gall of male (right), ex Eucalyptus microcarpa, Victoria, Long Forest, near Bacchus Marsh. Tanyscelis tripocula sp. n.: **f** galls of females, ex Eucalyptus cephalocarpa, Victoria, Macclesfield. Tanyscelis verrucula: **g** gall of female, ex Eucalyptus cephalocarpa, Victoria, Macclesfield **h** galls of males, ex Eucalyptus ?goniocalyx, Victoria, Bendigo. Tanyscelis villosigibba sp. n.: **i** gall of adult female, ex ironbark, Queensland, Dunmore State Forest. [Photographs by PJG except **a** from HMB photographic collection number 696A]

The morphological terms for Eriococcidae used in the descriptions follow those of Williams (1985), Miller and McKenzie (1967) and Afifi (1968). We use the term ‘vestibule’ for the inner end of a tubular duct, where the contents of the inner ductule empty into the main duct (as in Miller and McKenzie 1967), and the term 'cribriform plates' refers to sclerotic areas of cuticle that house aggregations of macrotubular ducts, from each of which, in live insects, a hollow filament of wax is produced. Illustrations of the adult females and first-instar nymphs of the new Australian species were prepared by NBH with a drawing tube and the Adobe programs Photoshop CS and Illustrator CS. Following the convention for scale insects, each figure displays the dorsal body surface on the left side of the page, and the ventral body surface on the right. In general, translucent pores, small setae, ducts and pores on the main drawing are not drawn to scale because otherwise they would be too small to be seen easily. Enlargements of cuticular features that are placed around the main drawing also are not drawn to scale. We have described the galls of the adult females of each species and, if available, also the gall of the male insects. As far as known, we have indicated the leaf surface that bears the gall orifice (the opening to the chamber housing the insect), but many Eucalyptus species have adult leaves that are pendulous and isobilateral (Chippendale 1988; Evans and Vogelmann 2006), and thus often it is difficult to distinguish the abaxial (lower) and adaxial (upper) leaf surfaces, especially from isolated leaves in museum collections. In addition, we have provided photographs of the galls of females and, if available, the galls of male insects for all but one species (Figs 1–3). These figures are placed together at the beginning of the descriptions to facilitate comparisons of the galls, which are useful for species determination.

This study draws extensively from the collections of P. J. Gullan (98 records), L. G. Cook (25 records), H. M. Brookes (25 records) and N. B. Hardy with PJG (19 records). The late Helen Brookes (formerly at the Waite Agricultural Research Institute, University of Adelaide, South Australia) acquired and curated a large collection of Coccoidea (including Opisthoscelis and Tanyscelis) during her career and, following her retirement in 1982, deposited this collection and associated notes and photographs in the Australian National Insect Collection (Upton 1997; Taylor and Keller 2008). Brookes also recorded biological and other information on cards filed by a Specimen Index Number that consisted of a number for the collection and an abbreviation of the year, for example, 44/65 was her 44^th^ collection for 1965 (Gullan and Williams in press). We have made use of her information on the biology and appearance of some Opisthoscelis and Tanyscelis species.

In the descriptions, we list the slide-mounted specimens that were examined, but most of these collections have associated dry gall material that is too numerous to record and list. Galls and associated slide-mounted insects are stored in the same collections. For many of the species, one to several slide-mounted specimens listed in the ‘Material examined’ are DNA voucher specimens of either L.G. Cook (e.g., LGC00101 for O. thurgoona) or NBH (e.g., NH104 for O. thurgoona) and all are deposited in ANIC (see below). The reliability of early (1890s to 1960s) identifications of eucalypt host plants is uncertain, but more recent records (1970s to present) are considered reliable. The classification of Brooker (2000) is used for taxonomic sections within Eucalyptus.

Depositories are abbreviated as follows: ANICAustralian National Insect Collection, CSIRO, Canberra, ACT, Australia; ASCUAgricultural Scientific Collections Unit, Orange Agricultural Institute, New South Wales, Australia; BMNHthe Natural History Museum, London, UK; BPBMBernice P. Bishop Museum, Honolulu, HI, USA; NMVMuseum of Victoria, Melbourne, Australia; NZACNew Zealand Arthropod Collection, Landcare Research, Auckland, New Zealand; QDPIQueensland Primary Industries and Fisheries, Indooroopilly, Queensland, Australia; SAMASouth Australian Museum, Adelaide; USNMthe United States National Collection of Coccoidea of the National Museum of Natural History, Smithsonian Institution, housed at the United States Department of Agriculture, Beltsville, Maryland. Collector and author names are abbreviated as follows: HMBH. M. Brookes, LGCL. G. Cook; WWFW. W. Froggatt; NBHN. B. Hardy; PJGP. J. Gullan.

The International Commission on Zoological Nomenclature (1999) requires lectotypes designated after 1999 to “contain an express statement of deliberate designation” (amended Article 74.7.3). We use the statement ‘here designated’ to fulfill this requirement. Lectotypes have been designated where a name lacks a holotype or lectotype and unambiguous syntypes have been identified. The purpose is to provide stability of nomenclature, and designation is done in a revisionary context in agreement with the amended Recommendation 74G of Article 74.7.3.

## Taxonomy

The key by Hardy and Gullan (2007) to adult females of genera of Eriococcidae found on Eucalyptus and Corymbia in Australia can be used to distinguish the adult female insects of Opisthoscelis and Tanyscelis from those of all other eriococcid genera on Eucalyptus. Species of Tanyscelis will key to Opisthoscelis in Hardy and Gullan (2007).

### Key to adult females of species of Opisthoscelis and Tanyscelis

**Table:** 

1.	Abdomen not tapered; anal ring on ventral body surface, usually invaginated, with ≥ 6 ring setae; fore and mid legs and antennae clearly multi-segmented, although often much reduced; cribriform plates or tight clusters of tubular ducts present or absent on dorsum; margins of posterior abdominal segments usually bearing fleshy projections that often bear spines or setae; galls always on leaves	2 (Opisthoscelis Schrader)
–	Abdomen tapered; anal ring poorly developed, without pores and with ≤ 6 minute setae, never invaginated, at apex of abdomen or on dorsal surface; fore and mid legs and antennae usually appearing unsegmented and always highly reduced; cribriform plates and tight clusters of tubular ducts absent from dorsum; marginal fleshy projections absent from abdomen (4 spine-tipped fleshy projections on abdominal segment VIII may be present); galls on leaves, stems or occasionally fruits	7 (Tanyscelis gen. n.)
2.	Posterior abdominal margin without fleshy projections; fore and mid legs well developed, all leg segments distinct	3
–	Posterior abdominal margin with fleshy projections, often as small segmental lobes; fore and mid legs reduced, some leg segments fused	4
3.	Antenna with 5 fleshy setae; dorsal cribriform plates heavily sclerotised, some on margin composed of > 10 ducts; hind tibia straight; hind tarsus similar to mid and fore tarsi, not expanded	Opisthoscelis tuberculata sp. n.
–	Antenna with 4 fleshy setae; dorsal cribriform plates weakly sclerotised, none composed of > 10 ducts; hind tibia with distal area of lateral margin concave; hind tarsus expanded, conspicuously larger than mid and fore tarsi	Opisthoscelis thurgoona sp. n.
4.	Marginal fleshy projections present on abdominal segments anterior to abdominal segment VII, each projection bearing 1–2 blunt or conical spines; dorsal cribriform plates absent	5
–	Marginal fleshy projections restricted to abdominal segment VII and VIII, each projection bearing > 2 spines; dorsal cribriform plates present	6
5.	Cuticle on dorsal head surface rugose and sclerotic; marginal projections on each abdominal segment, with each projection bearing conical spine(s); microducts absent; marginal fringe of elongate setae absent	Opisthoscelis serrata Froggatt
–	Cuticle on dorsal head surface not differentiated from rest of dorsum; marginal projections restricted to abdominal segments IV to VIII or V to VIII, with spines on projections blunt; microducts present; marginal fringe of elongate setae present	Opisthoscelis ungulifinis sp. n.
6.	Dorsal setae much smaller than marginal setae; marginal fringe of close-set conical setae distinct; eye dorsad of marginal fringe	Opisthoscelis beardsleyi sp. n.
–	Dorsal setae about as large as marginal setae; marginal fringe present but not pronounced; eye on margin	Opisthoscelis subrotunda Schrader
7.	Dorsum with shield of rugose sclerotic cuticle with 3 deep invaginations along midline; tubular ducts absent	Tanyscelis tripocula sp. n.
–	Dorsal derm membranous, or weakly sclerotic, without shield or deep invaginations; tubular ducts present or absent	8
8.	Dorsum or margin with humps or other evaginations	9
–	Dorsum and margin without humps or evaginations	13
9.	Eyes absent, replaced by a pair of fleshy evaginations	10
–	Eyes present, not mounted on fleshy evaginations	11
10.	One large papilliform evagination present on each side of head on anterior margin, each evagination > length of hind tarsus; other fleshy evaginations absent; coxa conical	Tanyscelis mollicornuta sp. n.
–	Papilliform evaginations on anterior margin of head < length of hind tarsus; additional evaginations present along dorsal midline plus thoracic submargin; coxa cylindrical	Tanyscelis grallator sp. n.
11.	Dorsum with three large humps, dorsal setae each mounted on swollen base; eye highly convex, base parallel-sided and perpendicular to body surface; hind tibia curved	Tanyscelis villosigibba sp. n.
–	Dorsal humps variable, dorsal setae without swollen bases; eye without parallel-sided base; hind tibia straight	12
12.	Macrotubular ducts present on dorsum; small humps present along midline on thorax and anterior abdominal segments; eye small (20–50 µm wide)	Tanyscelis spinosa (Froggatt)
–	Macrotubular ducts absent from dorsum; dorsum of mature females dominated by massive humps; eye large (60–85 µm wide)	Tanyscelis megagibba sp. n.
13.	Anal area with 4 stout spines; anal ring sclerotic, sometimes appearing horseshoe-shaped, with 6 fine setae	14
–	Anal area without 4 stout spines; anal ring poorly developed, unsclerotised	15
14.	Fleshy evaginations present immediately caudad of each spiracle; eye large (30–65 mm wide); small spines present along margin of head	Tanyscelis verrucula (Froggatt)
–	Fleshy evaginations near spiracles absent; eye small (15–25 mm wide); spines absent from head margin	Tanyscelis pisiformis (Froggatt)
15.	Hind tibia and tarsus fused, hind claw absent, tibia and tarsus forming robust sword-shaped segment; tubular ducts absent	Tanyscelis conica (Fuller)
–	Hind tibia and tarsus separate, hind claw reduced; tubular ducts present	16
16.	Stout spines present in band extending from anterior head margin to posterior spiracles; medial portions of dorsum with numerous conical fleshy projections, each with sclerotic spine at apex, several spines with ante-apical hair-like bristle	Tanyscelis convexa (Froggatt)
–	Stout spines absent; dorsum without conical fleshy projections bearing spines	17
17.	Eye large (45–123 mm wide); marginal setae short (longest setae ca 40 mm long), not forming conspicuous fringe	Tanyscelis maculata (Froggatt)
–	Eye small (20–45 mm wide); marginal setae long (longest setae ca 150 mm long), forming conspicuous fringe	Tanyscelis maskelli (Froggatt)

### Opisthoscelis

Schrader

Opisthoscelis Schrader 1863: 7. Type species: Opisthoscelis subrotundaSchrader 1863, subsequently designated by Fernald 1903: 46.
Ophistocelis ; Signoret 1868: 525. Misspelling of genus name
Ophiscelis ; Signoret 1869a: 834. Misspelling of genus name.
Opliscelis ; Signoret 1869a: 855, 872. Misspelling of genus name.
Ophistoscelis ; Signoret 1869b: 100. Misspelling of genus name.

#### Generic diagnosis.

##### Adult female and associated gall.

Galls on leaves; each typically globular, conical or hemispherical, rarely pit-like; with small circular, slit-like or fissured orifice/opening often on adaxial (upper) surface, but surface often difficult to determine in mature isobilateral leaves and galls typically opening on same surface on any one leaf. Body outline circular to elliptical; dorsum usually smaller than venter, especially at maturity, with whole of dorsum, a sclerotised part of it, or abdominal apex plugging gall orifice; body of mature female usually tightly fitting gall cavity. Abdomen not tapered. Vulva and anal opening ventral on posterior abdomen, with vulva between abdominal segments VII and VIII. Eyes on margin or dorsad of it. Antennae reduced, with ≤ 7 segments, segmentation often indistinct. Pair of broad frontal lobes posteromedial of antennae. Oral lobes membranous to sclerotic, often forming large circular pad around mouthparts. Tentorial box with aliform anterior extensions. Fore and mid legs varying from well developed to highly reduced, with some segmentation always apparent. Hind legs, on posterior of metathorax, sometimes appearing to be on anterior abdomen, large, always well developed, often elongate; ratio of length of trochanter + femur / length of tibia + tarsus approximately = 1:1; tibia-femur articulation functional; translucent pores present on at least tibia; trochanter with 2 or 3 campaniform sensilla on each side; claw digitules and tarsal digitules either well developed or highly reduced to absent. Anal opening surrounded by anal ring with ≥ 6 (range 6–20) setae; anal ring may be invaginated. Posterior abdominal segments usually with marginal fleshy projections (absent in Opisthoscelis thurgoona sp. n. and Opisthoscelis tuberculata sp. n.), each projection bearing spines or with a blunt sclerotic point. Marginal fringe of enlarged setae usually present (absent in Opisthoscelis serrata). Dorsal setae bristle-like to flagellate, minute to large, 4–148 µm long (stout conical setae present in Opisthoscelis subrotunda). Cribriform plates or tight clusters of tubular ducts either present or absent on dorsum. Microtubular ducts usually absent (present in Opisthoscelis ungulifinis sp. n.). Dorsal quinquelocular pores absent. Venter with macrotubular ducts present or absent; if present, with vestibule thin and sclerotic. Quinquelocular pores present on venter, at least around vulva and spiracles.

##### Adult male.

Antenna 10-segmented. Abdomen not elongated. Gland pouches present, each with pair of setae.

##### First-instar nymph.

Anterior margin of head incised at midline. Each spiracle with one trilocular pore next to opening. One submedial longitudinal row of dorsal setae on each side of body. Antennae 4-segmented, with 4 fleshy setae on apical segments.

#### Etymology.

The genus name is a Latinised combination of the Greek words *opisthen*, meaning behind, and *skelos*, meaning leg, and clearly refers to the long hind legs of the adult female because Schrader (1863: 6) diagnosed Opisthoscelis with one brief statement: “Where they have only two long posterior legs.” The name Opisthoscelis is treated as feminine.

#### Opisthoscelis beardsleyi

Hardy & Gullan sp. n.

urn:lsid:zoobank.org:act:F50EFB17-BBA7-4EBD-A6F3-4E3B93F2854D

Figs 1a,b,c 4 

##### Gall

(Fig. 1a,b,c).

###### Female.

On leaf. Gall opening circular, 0.3–0.4 mm wide, blocked by white wax and/or part of sclerotised posterior dorsum of female. Gall variable, usually 3–5 mm in diameter, side with opening subconical (Fig. 1a) or hemispherically rounded (Fig. 1b), with truncate apex, typically on adaxial surface; leaf glands enlarged.

###### Male.

On leaf. Similar to gall of female but smaller and more cylindrical, 2–3 mm high, basal diameter 1.5–2.0 mm, with circular opening and truncate apex ca 1 mm across (Fig. 1c), usually on adaxial leaf surface.

##### Adult female

(Fig. 3) (n = 47). Body outline circular, length 1.4–3.3 mm, greatest width 1.2–2.8 mm, abdomen not tapered, anal opening ventral, vulva as far cephalad as distal end of hind coxa. Eyes set well away from margin, each 20–60 µm wide. Antennal segmentation indistinct; each antenna 63–120 mm long. Frontal lobes each 110–330 µm long, 83–350 µm wide. Tentorial box 153–590 mm long; labium 73–125 mm long, 65–128 mm wide; pump chamber 17–22 µm long, 20–23 µm wide. Spiracles 63–170 mm long, 35–90 mm wide across atrium. Fore and mid legs small stumps, some segmentation apparent, 68–190 µm long; hind leg with coxa 200–440 µm long, trochanter + femur 260–510 µm long, tibia slightly curved, outer margin concave, 250–410 µm long, tarsus 70–180 µm long; claw and digitules present but reduced; translucent pores dense on both surfaces of hind tarsus, tibia and distal portion of femur; tibia-femur articulation functional. Anal opening 23–55 µm wide; anal ring 48–110 µm wide, bearing 10–16 setae, most anal ring setae with 1 or 2 pores near base; anal ring set within membranous invagination; area between anal ring and dorsal shield sclerotised in some specimens.

###### Dorsum.

Dorsal shield much smaller than venter in old specimens, of variably sclerotic cuticle 1.1–2.0 mm long, 1.0–2.7 mm wide, clearly delineated by marginal fringe of close-set spinose setae, each seta 25–70 µm long. A pair of fleshy caudal projections on each side of body; each medial projection with ca 4 spinose setae, each lateral projection (probably marginal lobe of abdominal segment VII) with usually 3 spinose setae. Dorsal setae mostly small and robust, 8–23 mm long, scattered over dorsum, a few large spinose setae similar to those in marginal fringe sometimes occur along submargin. Macrotubular ducts occurring in clusters of 1–25 ducts set within heavily sclerotic cribriform plate; shaft of each duct short, < 5 µm long; number of cribriform plates on dorsum varying from ca 10 to ca 40 on each side of body. Microtubular ducts absent. Quinquelocular pores absent.

###### Venter.

Oral lobes membranous. Setae 8–100 mm long, in transverse row or band across each abdominal segment, metathorax, and mesothorax, along margin of head, pro- and mesothorax; setae dense posterodorsal of anal ring. Macrotubular ducts absent. Quinquelocular pores 5–6 µm in diameter, in clusters around each spiracle and along margin of thorax and anterior abdominal segments.

**Figure 4.**
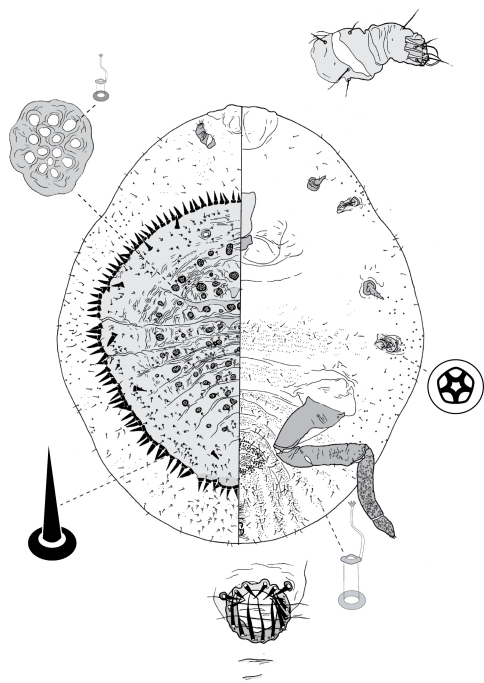
Adult female of Opisthoscelis beardsleyi Hardy & Gullan, sp. n.

##### Material examined.

###### Holotype of Opisthoscelis beardsleyi (here designated):

**AUSTRALIA: Victoria:** 1 adult female (2.3 mm long, 2.2 mm wide): ex gall, Eucalyptus goniocalyx, Tallangatta, Mt Granya, 26 May, 1976, PJG (ANIC).

###### Paratypes:

**AUSTRALIA: Victoria:** 13 adult females: same data as holotype (11 in ANIC, 1 in BMNH, 1 in USNM).

###### Additional material:

**AUSTRALIA: New South Wales:** 4 adult females: ex leaf galls, Eucalyptus sp., Coolangubra State Forest, Wog Wog site, 13 Mar., 1993, PJG (ANIC); 5 adult females: ex leaf galls, Eucalyptus nortonii, Old Tuggeranong Railway Station, -35.43°; 149.15°, 13 June, 1993, LGC (ANIC); 1 adult female: ex leaf gall, Eucalyptus sp., Tallong cemetery, -34.72°; 150.08°, 16 Oct., 1993, PJG (ANIC). **Victoria:** 3 adult females: ex leaf galls, Eucalyptus goniocalyx, Belgrave South, ca 0.5 km NNE of roundabout at Temple Road, on Belgrave–Hallam Road, -37.93°; 145.36°, 8 Feb., 2005, PJG and NBH, NH53, NH77 (ANIC, NMV); 12 adult females, 3 second-instar females: ex leaf galls, Eucalyptus viminalis, Cranbourne Botanic Gardens Annexe, 19 Oct., 1976, PJG (ANIC); 2 adult females: ex leaf galls, Eucalyptus viminalis, Cranbourne, Royal Botanic Gardens Cranbourne, Possum Gully Track, -38.13°; 145.28°, 9 Feb., 2005, PJG, NH33 (ANIC); 3 adult females: ex leaf galls, Eucalyptus goniocalyx, Dandenong, Doongalla Estate, The Basin, 8 May, 1977, PJG (ANIC); 6 adult females: ex leaf galls, Eucalyptus aromaphloia, Grampians Nat. Park, Victoria Valley, Glenelg River Road, W of Moora Moora Reservoir, -37.23°; 142.41°, 6 Feb., 2005, PJG and NBH, NH30 (ANIC, NMV); 7 adult females: ex galls, Eucalyptus aromaphloia, Grampians, Victoria Valley, 3.5 km N along Henham Track from Serra Road intersection, 18 Nov., 1976, PJG (ANIC); 19 adult females, 4 adult males: ex galls on leaves, Eucalyptus ovata, Lysterfield, Wellington Road, c. 0.5 km E of intersection with Lysterfield Road, 6 Feb., 1977, PJG (ANIC); 5 adult females: ex galls, Eucalyptus ovata, Lysterfield, Wellington Road, ca 0.5 km E of intersection with Lysterfield Road, 16 Feb,. 1979, PJG (ANIC); 2 adult females: ex leaf galls, Eucalyptus ovata, Lysterfield, Wellington Road, ca 1.9 km WNW of Belgrave–Hallam Road, -37.95°; 145.33°, 8 Feb., 2005, PJG and NBH, NH32 (ANIC); 10 adult females: ex leaf galls, Eucalyptus cephalocarpa, Macclesfield, Kirkpatrick’s Road, 17 Oct., 1977, PJG (ANIC); 1 adult female: ex leaf gall, Eucalyptus ?goniocalyx, Melbourne, North Warrandyte, corner of Overbank Road and Glynns Road, -37.73°; 145.20°, 14 Feb,. 2005, PJG and NBH, NH49 (ANIC); 1 adult female: ex leaf gall, Eucalyptus cypellocarpa, near Apollo Bay, Beacon Hill, ca 3 km N of Great Ocean Road, on Skenes Creek Road, -38.72°; 143.71°, 11 Feb., 2005, PJG and NBH, NH51 (ANIC); 1 adult female: ex leaf gall, Eucalyptus ?goniocalyx, NW of Bunyip, Jefferson Road off Princes Freeway, S side, -38.08°; 145.69°, 13 Feb., 2005, PJG and NBH, NH50 (ANIC).

##### Comments.

The adult female of Opisthoscelis beardsleyi is most similar to that of Opisthoscelis subrotunda in that both species have dorsal cribriform plates, a marginal fringe of spinose setae, and ≥ 2 spinose setae mounted on each caudal fleshy projection or lobe (these projections poorly developed in some populations of Opisthoscelis beardsleyi). Adult females of Opisthoscelis subrotunda can be recognised by having the dorsal derm densely beset with minute papillae (found in some species of Tanyscelis but no other Opisthoscelis species) and large, spinose dorsal setae (dorsal setae minute in Tanyscelis, or bristle-like in other species of Opisthoscelis).

Galls of adult females of Opisthoscelis beardsleyi may be conical (Fig. 1a) or rounded with either a truncate (Fig. 1b) or convex apex. Adult females vary in the number of cribriform plates and enlarged setae on the dorsal submargin as well as the extent to which the caudal projections are developed. It is possible that the material we have listed under the name Opisthoscelis beardsleyi constitutes multiple species, but we have not been able to detect any clear patterns in morphological variation over geography or host use. Therefore, we have restricted the type material to a single collection. Specimens of Opisthoscelis beardsleyi have been collected only from southeastern Australia, from eucalypt species in the Symphyomyrtus section Maidenaria.

##### Etymology.

This species is named in honour of the late Dr. Jack Beardsley, who was an expert on the systematics of parasitic wasps and scale insects. He spent most of his entomological career at the University of Hawaii, but began studies on Australian gall-inducing eriococcids during a research sabbatical spent in Victoria in 1971–1972. He was interested especially in pit-inducing and cryptic bark-living eriococcids and amassed a substantial collection from Victoria, including some specimens of Opisthoscelis and Tanyscelis.

#### Opisthoscelis serrata

Froggatt

Figs 1d,e,f 5

Opisthoscelis serrata Froggatt 1894b: 346–347.

##### Gall

(Fig. 1d,e,f).

###### Female.

On leaf. Height 2.4–7.0 mm, width 2.5–6.6 mm, length of basal attachment 2.4–4.0 mm. Gall opening slit-like ca 2 mm wide, on abaxial leaf surface. Gall spherical, sometimes with a tapered point (Fig. 1d), opening on opposite side of leaf. Mature female filling gall cavity (Fig. 1e).

###### Male.

On leaf. Gall opening oblong, surrounded by a slightly raised lip (Fig. 1f), on abaxial leaf surface; opposite side of leaf with small globose swelling.

##### Adult female

**Figure 5.**
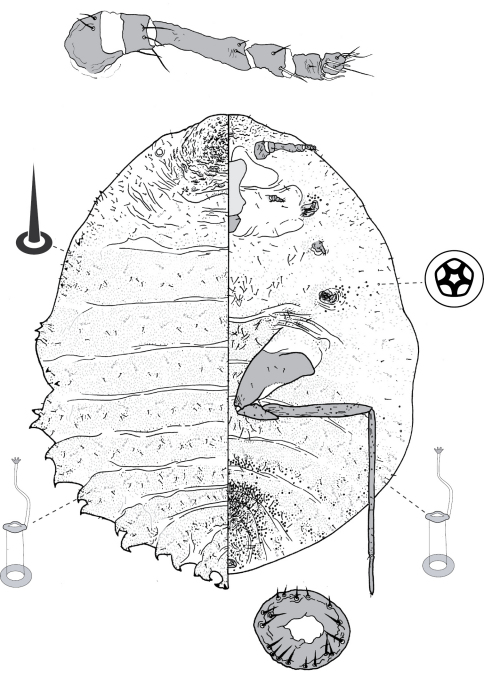
Adult female of Opisthoscelis serrata Froggatt.

(Fig. 5) (n = 21). Body outline ovate, length 2.3–5.0 mm, greatest width 1.8–4.3 mm; abdomen about as long as head + thorax. Eyespots set well away from margin, each 28–90 mm wide. Antennae 6-segmented, each 290–490 mm long. Frontal lobes may be difficult to discern, each 225–470 µm long, 150–490 µm wide. Tentorial box 285–480 mm long. Labium 75–155 mm long, 100–168 mm wide. Pump chamber 25–28 µm long, 23–30 µm wide. Spiracles 68–195 mm long, 48–85 mm wide across atrium. Fore and mid legs reduced but segmented, 43–160 µm long. Hind legs slender and elongate; coxa 300–590 µm long; trochanter + femur 480–940 µm long; tibia straight, 460–1000 µm long; tarsus 180–280 µm long; claw and digitules present but reduced; few translucent pores scattered on femur, tibia and tarsus; femur-tibia articulation functional. Anal opening ventral, set in shallow membranous invagination, 30–55 µm wide; anal ring plate-like, 63–128 µm wide, with 6–18 setae.

###### Dorsum.

Head with shield of rugose, sclerotic cuticle, 600–1000 µm long, 450–1100 µm wide; each side of abdomen with segmental pairs of marginal spines, each spine at end of fleshy projection, these larger on posterior segments; each thoracic segment with a few similar structures along the margin; marginal fringe of elongate setae absent. Derm densely beset with microtrichia. Dorsal setae minute, 4–8 mm long; scattered over dorsum. Macrotubular ducts 12–15 µm long, rim of dermal orifice 5 µm wide; ducts in a transverse row across each body segment. Microtubular ducts absent. Quinquelocular pores absent.

###### Venter.

Oral lobes membranous. Ventral setae 5–115 mm long, in transverse row across each abdominal segment, a medial to submedial cluster on each thoracic segment, scattered along margin, longer setae found in medial areas. Macrotubular ducts similar to those on dorsum, scattered along margin, in a transverse row across each of abdominal segments II–IV. Quinquelocular pores 5 mm in diameter, in cluster around each spiracle, along margin of abdomen, in a transverse band across each posterior margin of abdominal segments IV–VIII.

##### Material examined.

###### Lectotype of Opisthoscelis serrata (here designated):

**AUSTRALIA: Victoria:** 1 adult female (damaged, ca 3.0 mm mm long, 2.7 mm wide), on slide with another adult female, lectotype farthest from data label; original label: “Opisthescelis[sic] serrata / ♀ galls on Euc. Adult in / spirit. Slide in coll. / Bendigo W.W.F. / Damage in Box 284” ASCTHE101344 (ASCU).

###### Paralectotypes:

**AUSTRALIA: Victoria:** 1 adult female, same data and slide as lectotype (ASCU); dry galls (6 of female and 4 of male), all parasitised or empty, with printed label: “3046 E / GALL MAKING COCCIDS, / Opisthoscelis serrata, Frogtt. / Female galls upon Eucalyptus sp. / Bendigo, Victoria.” ASCT00004861 (ASCU).

The lectotype and associated paralectotype are in poor condition and were mounted from the spirit collection in ASCU by PJG in 1985; additional females, in poor condition, remained in the vial; no original WWF slide-mount was located.

###### Additional material:

**AUSTRALIA: New South Wales:** 5 adult females, 5 second-instar females: ex galls on leaves, Eucalyptus largiflorens, Kinchega National Park, 2 July, 1984, J. M. Smith (ANIC). **South Australia:** 9 adult females: ex galls, Eucalyptus microcarpa, Aldinga Beach, 2 Oct., 1965, HMB, Specimen Index No. 44/65 (ANIC); 5 adult females, 6 second-instar females: ex globular galls on leaves, Eucalyptus odorata [may be misidentification of Eucalyptus microcarpa], Wilmington, 7 Sep., 1982, F. D. Morgan, Specimen Index No. 19/82 (ANIC). **Victoria:** 7 adult females: ex galls on leaves, Eucalyptus ?microcarpa, ca 10 km S of Nagambie, just E of railway line, -36.37°; 145.17°, 7 Feb., 2004, PJG (ANIC); 2 adult females: ex galls on leaves, Eucalyptus microcarpa, ca 10 km S of Nagambie, on road to Avenel, near railway line, -36.38°; 145.17°, 30 Jan., 2005, PJG, NH26 (ANIC); 2 adult females: ex galls on leaves, Eucalyptus melliodora, ca 9 km N of Nagambie, on Goulburn Weir Road, -36.72°; 145.18°, 30 Jan., 2005, PJG, NH24 (ANIC); 4 adult females: ex galls on leaves, Eucalyptus polyanthemos, Melbourne, North Warrandyte, corner of Overbank Road and Glynns Road, -37.73°; 145.20°, 14 Feb., 2005, PJG & NBH (ANIC, NMV); 2 adult females: Eucalyptus polyanthemos, Melbourne, Warrandyte, behind 134 Brackenbury Street, 7 May, 1977, PJG (ANIC).

##### Comments.

Adult females of Opisthoscelis serrata can be recognised easily by (1) the shield of rugose sclerotic cuticle on the dorsal surface of the head, and (2) the pair of marginal spine-tipped fleshy projections on each side of each abdominal segment. Both of these features are unique amongst Opisthoscelis and Tanyscelis species. Opisthoscelis serrata is known only from eucalypt species in the section Adnataria. There is some variation in the shape and size of the gall of the adult female, which is basically globular. Galls on leaves of Eucalyptus largiflorens and Eucalyptus polyanthemos protrude as a sphere on one side of the leaf, although some galls from leaves of Eucalyptus polyanthemos at Warrandyte have a pointed apex (Fig. 1d). In contrast, galls from the leaves of Eucalyptus melliodora and Eucalyptus microcarpa from near Nagambie protrude equally from both leaf surfaces and the females are smaller than those from most other localities. Females from all galls look similar, although differing in size.

#### Opisthoscelis subrotunda

Schrader

Figs 1g,h,i 6 7

Opisthoscelis subrotunda Schrader 1863: 7.
Opisthoscelis globosa Rübsaamen 1894: 214; synonymy by Froggatt 1894a: 209.

##### General.

Schrader (1863) described and illustrated Opisthoscelis subrotunda based on insects and galls from an unidentified Eucalyptus sp. collected in New South Wales. Schrader’s collection was destroyed on July 30, 1943, during WWII according to Ew. Weidner in personal communication to PJG (Gullan 1984a). Rübsaamen (1894) described a number of Australian gall-inducing insects, including Opisthoscelis globosa from Eucalyptus in New South Wales, while his address was “Königlichen Museum für Naturkunde zu Berlin”. We contacted Museum für Naturkunde Leibniz-Institut für Evolutions- und Biodiversitätsforschung an der Humboldt-Universität zu Berlin, but were told that there was no Opisthoscelis material from Rübsaamen in that collection, and thus we believe that Rübsaamen’s material also was destroyed during WWII, as suggested by Gullan (1984a). The synonymy of Opisthoscelis subrotunda and Rübsaamen’s Opisthoscelis globosa was proposed by Froggatt (1894a) but not accepted by Hoy (1963); subsequent authors have followed Hoy, although Miller and Gimpel (2000) stated that the status of these two species needed further study. Here we accept Froggatt’s (1894a) synonymy of these two species based on the perfect match of Schrader’s and Rübsaamen’s descriptions and illustrations of the insects and their galls. There is only one species in New South Wales that exactly matches Schrader’s and Rübsaamen’s descriptions. Thus we are certain of the identity of Opisthoscelis subrotunda and consequently have not designated a neotype.

##### Gall

(Fig. 1g,h,i).

###### Female.

On leaf. Gall globose (Fig. 1g), surface punctate, leaf glands enlarged on gall. Gall height 4.0–8.7 mm, width 4.2–9.5 mm, length of basal attachment 3.0–6.8 mm; wall of gall at least 2 mm thick in most mature galls. Gall opening variable, slit-like, cruciform (Fig. 1h) or round, sometimes with “lips” projecting, 0.1–2.5 mm wide; opening on abaxial or adaxial surface, but usually on same surface on any one leaf or plant. Mature female fills gall cavity with her abdominal apex directed towards and plugging gall orifice.

###### Male.

On leaf. Gall conical (Fig. 1i), distal margin entire, opening oblong to round, 0.5–2.2 mm wide. Gall height 1.4–4.1 mm, width 1.1–3.6 mm, length of basal attachment 1.3–3.7 mm.

##### Adult female

**Figure 6.**
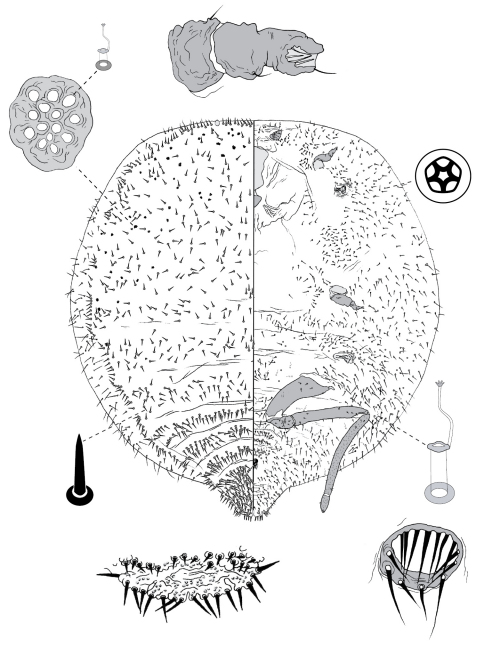
Adult female of Opisthoscelis subrotunda Schrader

(Fig. 6) (n = 30). Body outline ovate, length 2.1–4.8 mm, greatest width 1.5–4.0 mm; abdomen about as long as head + thorax. Eyespots on margin, each 40–60 mm wide. Antennal segmentation poorly developed; each antenna 50–160 mm long. Frontal lobes sometimes difficult to discern, each 140–280 µm long, 100–240 µm wide. Tentorial box 260–560 mm long. Labium 70–140 mm long, 70–160 mm wide. Pump chamber 26–33 µm long, 28–33 µm wide. Spiracles 90–180 mm long, 50–120 mm wide across atrium. Fore and mid legs reduced but segmented, 115–360 µm long. Hind legs slender and elongate; coxa 370–640 µm long; trochanter + femur 520–950 µm long; tibia 420–1020 µm long; tarsus 110–170 µm long; claw and digitules present but reduced; translucent pores on both surfaces of tibia and tarsus; femur-tibia articulation functional. Anal opening ventral, set in shallow membranous invagination, 38–83 µm wide; anal ring 65–120 µm wide, with 8–16 setae.

###### Dorsum.

Delineated by fringe of spinose setae along margin, each seta similar to those on rest of dorsum; cauda composed of two truncate fleshy projections on each side of body, each bearing ca 4–6 spinose setae. Derm variously sclerotic, densely beset with minute papillae. Dorsal setae spinose, 20–138 mm long, with stout, or swollen bases. Microtubular ducts absent. Quinquelocular pores absent.

###### Venter.

Oral lobes membranous. Venter hirsute, each seta 23–163 mm long, absent from medial areas of head and pro- thorax, plus antero-medial portion of mesothorax. Macrotubular ducts 10–12 µm long, dermal orifice with rim 5 µm wide; ducts restricted to abdomen, scattered. Quinquelocular pores 5–8 mm in diameter; clustered around each spiracle, and on medial areas of abdomen.

##### First-instar nymph

**Figure 7.**
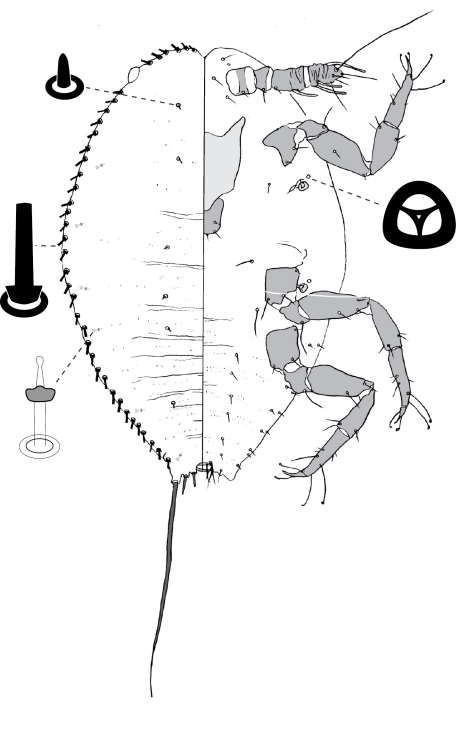
First-instar nymph of Opisthoscelis subrotunda Schrader.

(Fig. 7) (n = 4). Body outline elliptical, anterior margin incised at midline; length 288–308 µm, greatest width 180–198 µm. Eyespots on margin, each 10–11 mm wide. Antennae 4-segmented, each ca 75 mm long. Tentorial box 55–63 mm long. Labium 25–28 mm long, 28–33 mm wide. Spiracles 13–19 mm long, 8 mm wide across atrium. Each leg: coxa 23–28 µm long, with 5 setae; trochanter + femur 60–66 µm long, trochanter with 4 setae, femur with 4 setae; tibia 38 µm long, with 4 setae; tarsus 38–40 µm long, with 4 setae; claw 15–18 µm long; tarsal digitules capitate, unequal length, short digitule 23–24 µm long, long digitule 28–33 µm long, claw digitules capitate, each 15–18 µm long. Anal ring 15–16 µm wide, with 6 setae, each ca 13–18 µm long. Apical seta 135–183 µm long.

###### Dorsum.

Derm membranous. Dorsal setae ca 3 mm long; arranged in submedial longitudinal row on each side of body, 1 seta on each side of head, each thoracic segment, and each or any of abdominal segments I–III and V. Microtubular ducts 4 µm long, on each side of body: 1 duct on submargin of each side of each thoracic segment plus usually each of abdominal segments I, V and VIII. Marginal setae cylindrical, with hemispherical base with diameter > width of cylinder, distal edge of base flat, each marginal seta 2–5 µm long; each side of body with ca 6 setae between midline and eyes, 9 on prothorax, 5 on mesothorax, 3 on metathorax, 2 on each of abdominal segments I–VII, and on abdominal segment VIII 1 lateral and 2 medial of apical seta (these 3 probably homologous to anal lobe setae), with setae medial of apical seta robust, each with truncate apex.

###### Venter.

Setae hair-like, 2–23 mm long, each side of body with 3 setae medial of scape, 1 seta medial of each coxa, 3 longitudinal rows on abdomen, each row with 1 seta on each of abdominal segments II–VII; suranal and ventral lobe setae hair-like, each 18–25 µm long. Trilocular pores 2 µm in diameter, 1 pore near each spiracle.

##### Material examined.

**AUSTRALIA: Australian Capital Territory:** 3 adult females: Eucalyptus sp., Canberra, Bruce, 23 Sep., 1972, H. J. Banks (ANIC). **New South Wales:** 1 adult female: ex leaf gall, Eucalyptus blakelyi, 26 km N of Canowindra, 14 km S of Cudal, -33.41°; 148.69°, 13 Aug., 2004, NBH and PJG, NH21 (ANIC); 13 adult females: ex galls on leaves, Eucalyptus longifolia, 8.5 km SSE Moruya, Bingie Road, -35.97°; 150.12°, 7 Jan., 1992, PJG (ANIC); 5 adult females, 3 second-instar females, 2 first-instar nymphs: ex galls on leaves, Corymbia gummifera, Bundeena, roadside, 14 Feb., 2004, C. A. M. Reid and PJG, NH84 (ANIC); 5 adult females: ex leaf, Eucalyptus longifolia, Congo, 4 Jan., 1992, PJG (ANIC); 2 adult females: ex galls on leaves, Eucalyptus sp., Congo, Congo Road, -35.95°; 150.15°, 8 Jan., 1992, PJG (ANIC); 10 adult females: Sydney (ASCU); 1 adult female: ex leaf gall, Eucalyptus sp., Toolom, near Toolom Falls on edge of Yabbra State Forest, -28.52°; 152.53°, 22 Aug., 2004, NBH and PJG, NH23 (ANIC); 7 adult females (2 slides): Eucalyptus robusta, Sydney Harbour, W. W. Froggatt #1868 (ANIC); 4 adult females (2 slides): Eucalyptus resinifera, Taree, WWF #1941 (ANIC); 1 adult female: Eucalyptus sp., Hay, 1919, WWF #299 (BMNH); 1 adult female: Eucalyptus robusta, Sydney Harbour, 20 Dec., 1929, WWF (BMNH). **Northern Territory:** 5 adult females, 13 adult males: Eucalyptus camaldulensis var. obtusa, ca 120 km W of Alice Springs, Ormiston Gorge, 29 May, 1977, PJG (ANIC); 4 adult females: ex galls on leaves, Eucalyptus camaldulensis, Serpentine Gorge Nature Park, 27 May, 1992, PJG and P. S. Cranston (ANIC). **Queensland:** 1 adult female: ex gall, Eucalyptus resinifera, on road from Paluma to Hidden Valley, -19.02°; 146.12°, 30 Oct., 2003, LGC and M. D. Crisp, LGC00066 (ANIC); 4 adult females: Eucalyptus rostrata (now Eucalyptus camaldulensis), Barakula, 13 Apr., 1939 (QDPI); 1 adult female: Eucalyptus rostrata (now Eucalyptus camaldulensis), Barakula, Nov. 1939, No, 784 (QDPI); 5 adult females: Eucalyptus crebra, Clermont, Nov. 1939 (QDPI); 5 adult females: Eucalyptus melliodora or Eucalyptus rostrata (now Eucalyptus camaldulensis), Emu Vale, 8 Feb., 1939, No. SC394 (QDPI); 5 adult females: on leaves, Eucalyptus rostrata (now Eucalyptus camaldulensis), Emu Vale, 15 Mar., 1939 (QDPI); 3 adult females: Eucalyptus propinqua, Monto, 9 Nov. 1938, No. SC375 (QDPI); 3 adult females: Eucalyptus saligna, Pechey, 5 Mar., 1937, INSECOLL 0-067151, 0-067152 (QDPI); 2 adult females and ca 25 first-instar nymphs: Eucalyptus pilularis, Pechey, May 1938, No. SC326 (QDPI); 3 adult females: Eucalyptus tereticornis, Redland Bay, 9 Apr., 1939, No. 785 (QDPI); 1 adult female: Eucalyptus grandis, Tamborine, 20 Jan., 1954, INSECOLL 0-067167 (QDPI). **Victoria:** 1 adult female: ex gall on leaf, Eucalyptus camaldulensis, 27 km E of Shepparton, nr Midland Hwy, 8 Feb., 2004, PJG, LGC00099 (ANIC); 4 adult females: ex galls on leaves, Eucalyptus camaldulensis, Bundoora, La Trobe University, Wildlife Reserves off Ring Road, -37.72°; 145.05°, 14 Feb., 2005, PJG and NBH, NH43 (ANIC, NMV); 7 adult females: Eucalyptus mcintyrensis (= ? Eucalyptus camaldulensis or a hybrid), Glenthomson, 14 Feb., 1964, HMB, Specimen Index No. 08/64 (ANIC); 1 adult female: ex leaf gall, Eucalyptus camaldulensis, Mildura, River Road near Lock 11, Murray River, -34.17°; 142.16°, 4 Feb,. 2005, NBH and PJG, NH75 (ANIC); 1 adult female: ex globose gall on leaf, Eucalyptus ?camaldulensis, Shepparton, near Broken River, off Lincoln Drive, close to Goulburn Valley Hwy, -36.40°; 145.39°, 30 Jan., 2005, PJG, NH72 (ANIC); 1 adult female: ex globose leaf gall, Eucalyptus ?microcarpa, Shepparton, near Goulburn River, off Tom Collins Drive, -36.39°; 145.39°, 29 Jan., 2005, PJG, NH71 (ANIC). **Western Australia:** 8 adult: ex galls on leaves, Eucalyptus camaldulensis, Winjana Gorge Nat. Park, bank of Lennard River, -17.42°; 124.95°, 29 Apr., 1992, PJG (ANIC). **PAPUA NEW GUINEA:** 8 adult females, 20 first-instar nymphs: ex galls on leaves, Eucalyptus alba, Central Province, Sogeri, -9.43°; 147.58°, 10 Apr., 1980, H. Roberts (ANIC, BMNH).

##### Comments.

Adult females of Opisthoscelis subrotunda most closely resemble those of Opisthoscelis beardsleyi [see comments under Opisthoscelis beardsleyi]. Specimens of Opisthoscelis subrotunda have been collected from six Australian states or territories as well as Papua New Guinea. This is the only species of Opisthoscelis or Tanyscelis to have been collected outside of Australia. Opisthoscelis subrotunda also appears to have a much broader host range than is typical for species of Opisthoscelis and Tanyscelis. Specimens have been collected from Corymbia in addition to hosts in the Eucalyptus sections Adnataria, Exsertaria, Latoangulatae, and Similares (all in subgenus Symphyomytrus). A common host of this species is the river red gum, Eucalyptus camaldulensis, which has the widest natural distribution of any eucalypt species and seven subspecies (Chippendale 1988; McDonald et al. 2009). The length of the dorsal setae, and the size of the cribriform plates of the adult females of Opisthoscelis subrotunda vary, but in general, the morphology is remarkably homogeneous across samples. Molecular data from collections of fresh material from across the geographic range of the taxon would be required to determine if cryptic species are present.

#### Opisthoscelis thurgoona

Hardy & Gullan sp. n.

urn:lsid:zoobank.org:act:D2BC31B8-0355-4D1C-8F20-2E7DEFC47EEC

Figs 1j 8 

##### Gall

(Fig. 1j).

###### Female.

On leaf. Immature gall (housing yellow nymph with a red dorsal “keel”) shallowly conical to hemispherical on orifice side (Fig. 1j), with gall surface whitish green to reddish; almost hemispherical on opposite side of leaf, tissue reddish green with distinct oil glands on surface. Gall height on orifice side ca 1 mm, ca 3 mm on opposite side, width 4–6 mm. Gall opening on either abaxial or adaxial leaf surface, slit-like, 0.5–1.0 mm long. Mature gall with tissue surrounding orifice brown and necrotic.

###### Male.

Not known.

##### Adult female

**Figure 8.**
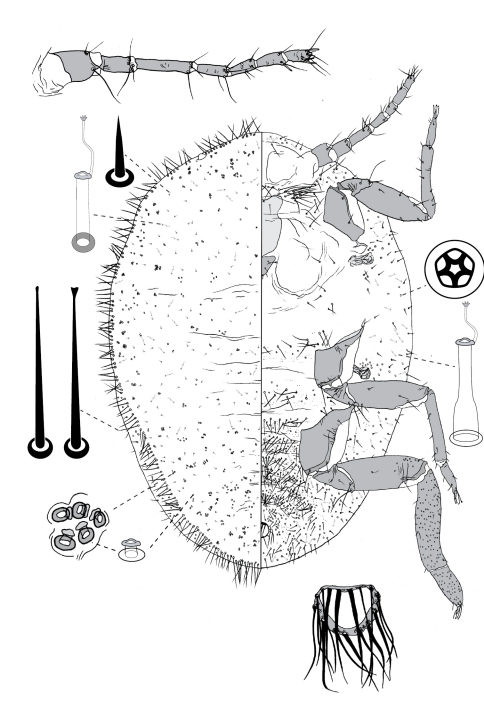
Adult female of Opisthoscelis thurgoona Hardy & Gullan, sp. n.

(Fig. 8) (n = 6). Body ovate, length 2.1–3.4 mm, greatest width 1.5–2.8 mm; margin without lobes or indentations. Eyes on margin, each 38–55 µm wide. Antennae well developed, 7-segmented; each antenna 530–890 mm long; segment I with ca 3 hair-like setae; II with 5–9 hair-like setae; III with 4–8 hair-like setae; IV 4–6 hair-like setae; V with 1–4 hair-like setae; VI with 3 or 4 hair-like setae + 1 fleshy seta; VII with ca 6 hair-like setae + 3 fleshy setae. Frontal lobes each 300–310 µm long, 125–220 µm wide. Tentorial box 200–300 mm long; labium 80–150 mm long, 95–170 mm wide; pump chamber 15 µm long, 15–16 µm wide. Spiracles 100–130 mm long, 50–73 mm wide across atrium. All legs well developed, hind legs larger than fore or mid legs: fore leg with coxa 230–360 µm long, trochanter + femur 345–530 µm long, tibia 235–400 µm long, tarsus 100–140 µm long; mid leg with coxa 310–430 µm long, trochanter + femur 370–580 µm long, tibia 260–450 µm long, tarsus 110–150 µm long; hind leg with coxa 350–500 µm long, trochanter + femur 460–670 µm long, tibia slightly curved, outer margin concave, 380–580 µm long, tarsus 150–240 µm long; each leg with claw 23–40 µm long; translucent pores dense on hind tarsus and tibia, a few scattered on distal portion of femur; femur-tibia articulation functional; tarsal and claw digitules distinctly expanded at apices. Anal opening 65–84 µm wide, on ventral body surface, surrounded by sclerotic anal ring 85–104 µm wide bearing 14–18 setae, base of each seta surrounded by small pores.

###### Dorsum.

Delineated by fringe of elongate setae, each seta 50–180 long, with blunt to truncate apex. Derm membranous. Dorsal setae minute, 5–9 mm long, scattered. Macrotubular ducts in two forms: (i) singly or in clusters of 2–8 ducts, cuticle of each cluster variously sclerotic, each duct short, ca 5 µm long, inner ductule not detected, scattered over dorsum, dense on head, posterior abdominal segments and margin of anterior abdominal segments and thorax; and (ii) single, larger ducts, 14–16 mm long, with dermal orifice with a rim 3.5–5.0 mm wide, scattered over dorsum, scarce or absent from posterior abdominal segments. Microtubular ducts absent. Quinquelocular pores absent.

###### Venter.

Oral lobes membranous. Setae 13–103 mm long, in a transverse row across each abdominal segment, scattered along margin of head and thorax, on medial areas of meso- and metathorax, and a transverse band of elongate (up to 170 µm long) setae posterior of frontal lobes. Macrotubular ducts with distal (near vestibule) end of shaft constricted, about same size as larger ducts on dorsum. Quinquelocular pores 5 µm in diameter, overlapping in distribution with ventral setae, with a cluster around each spiracle, but most dense medially on posterior abdominal segments.

##### Material examined.

###### Holotype of Opisthoscelis thurgoona (here designated):

**AUSTRALIA: New South Wales:** 1 adult female (3.4 mm long, 2.8 mm wide):ex leaf gall, Eucalyptus melliodora, 2 km NE of Thurgoona, Ettamogah Road, -36.03°; 146.98°, 8 Feb., 2004, PJG (ANIC).

###### Paratypes:

**AUSTRALIA: New South Wales:** 2 adult females: same data as holotype, NH104, LGC00101 (ANIC); 14 adult females, 1 second-instar female, 27 first-instar nymphs: ex galls on leaves, Eucalyptus melliodora, 1 km N of Thurgoona, Table Top Road, -36.05°; 146.98°, 30 Dec., 1991, PJG (ANIC, except 1 adult female in BMNH, 1 adult female in USNM).

##### Comments.

Adult females of Opisthoscelis thurgoona are unusual amongst Opisthoscelis and Tanyscelis species in having each leg well developed, and 7 clearly separate antennal segments. These features, in combination with the ventral anal opening, with a sclerotic anal ring bearing numerous setae, each of which is surrounded at the base by a number of minute pores, give the adult female of Opisthoscelis thurgoona the resemblance of a species of Lachnodius. Morphological clues of the closer relationship between Opisthoscelis thurgoona and the other Opisthoscelis species (recovered by analysis of DNA sequence data (NBH, unpublished data)) are the presence of macrotubular duct clusters on the dorsum (possibly homologous to the cribriform plates found in Opisthoscelis subrotunda and Opisthoscelis beardlseyi) and only 4 fleshy setae on each antenna (5 on species of Lachnodius). Opisthoscelis thurgoona is known only from a single roadside location, 1 km north of Thurgoona near Albury in New South Wales. Opisthoscelis thurgoona is most similar to Opisthoscelis tuberculata (described as new below) and the two species are compared under the comments for the latter.

Live eggs of Opisthoscelis thurgoona are laid in the gall cavity and are pink to orange in colour, whereas newly hatched first-instar nymphs are orange. The chromosome number is 2n = 18 (LGC, unpublished data).

##### Etymology.

The species name is taken from the type locality. It is a noun in apposition.

#### Opisthoscelis tuberculata

Hardy & Gullan sp. n.

urn:lsid:zoobank.org:act:F89DC70B-882F-4708-8C04-0B8C73E9C533

Figs 1k 9 

##### Leaf pit

(Fig. 1k).

###### Female.

On leaf. Pit-shaped gall, opening round to oblong, 2.0–3.0 mm wide, surrounded by raised lip, ca 0.2 mm high, apparently on adaxial leaf surface; gall hemispherical on side of leaf opposite opening, 1.0–1.5 mm high.

###### Male.

Not known.

##### Adult female

**Figure 9.**
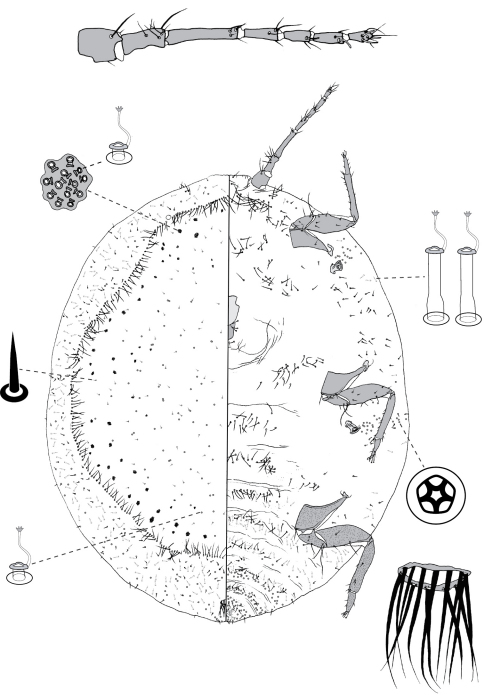
Adult female of Opisthoscelis tuberculata Hardy & Gullan, sp. n.

(Fig. 9) (n = 4). Body broadly oval in outline, length 2.0–4.0 mm, greatest width 1.4–3.2, venter larger than dorsum. Eyes each 40–50 um wide, slightly ventral of line of marginal setal fringe. Antennae 7-segmented, moderately long (0.72–1.10 mm) and slender; with segment III longest (180–290 µm) and segment VII shortest (70–95 µm); segment I with 3 hair-like setae; II with 7–11 hair-like setae; III with 8–12 hair-like setae; IV 5–7 hair-like setae; V with 4–6 hair-like setae + 1 fleshy seta; VI with 3 or 4 hair-like setae + 1 fleshy seta; VII with ca 6 hair-like setae + 3 fleshy setae. Frontal lobes difficult to discern, each perhaps 200–280 µm long, 110–120 µm wide. Tentorial box 125–200 mm long; labium 100–150 mm long, 120–140 mm wide, 2-segmented with basal segment indicated by presence of setae; pump chamber 20–27 µm long, 17–20 µm wide. Spiracles 90–140 mm long, 55–95 mm wide across atrium. All legs well developed, hind legs larger than fore or mid legs: fore leg with coxa 240–400 µm long, trochanter + femur 370–550 µm long, tibia 280–440 µm long, tarsus 110–160 µm long; mid leg with coxa 270–450 µm long, trochanter + femur 380–580 µm long, tibia 280–460 µm long, tarsus 110–150 µm long; hind leg with coxa 320–520 µm long, trochanter + femur 400–620 µm long, tibia straight, 310–500 µm long, tarsus 120–180 µm long; each leg with claw 35–45 µm long; small translucent pores scattered on both surfaces of all hind-leg segments, densest on tibia; femur-tibia articulation functional; tarsal and claw digitules distinctly expanded at apices. Anal opening 53–75 µm wide, on ventral body surface, surrounded by sclerotic anal ring 68–100 µm wide bearing 12–14 setae, each 150–175 µm long with setal base surrounded by 4–10 minute pores.

###### Dorsum.

Delineated by unbroken fringe of moderately dense setae, with approximately 140–170 moderately slender bluntly rounded or minutely capitate setae per side, setae each about 80–165 µm long on abdomen, somewhat shorter anteriorly, 65–100 µm long. Derm membranous. Dorsal body setae sparsely scattered, short (ca 7–15 µm long) and stout, with acute apex. Macrotubular ducts of one kind and size: short, ca 4–7 µm long, with a rim 3–4 µm wide; sparsely scattered singly or in pairs on central part of dorsum, but aggregated into distinct groups on peripheral areas, aggregations increasing in size toward margin, outer ones mostly containing about 10–25 ducts, maximum 30; duct aggregations borne on small, roughly oval to circular, sclerotised tubercles (cribriform plates). Microtubular ducts absent. Quinquelocular pores absent.

###### Venter.

Oral lobes membranous. Ventral body setae sparse, mostly 50–100 µm long, up to 150 µm long on head behind frontal lobes, shorter (20–50 µm long) in lateral pore band. Macrotubular ducts slender, inner portion dilated, 15–17 µm long, 2.0–2.5 µm wide, with poorly sclerotised rim 2.5–3.5 µm in diameter; ducts distributed in a moderately dense peripheral band, ducts distributed evenly across abdominal segments III–VI, but apparently absent on segments immediately in front of and behind vulva where disc pores are most numerous. Quinquelocular pores 5 µm in diameter, mostly confined to abdominal segments V–VIII anterior to anal ring, but a few (12–20) associated with each spiracle.

##### Material examined.

###### Holotype of Opisthoscelis tuberculata (here designated):

**AUSTRALIA: New South Wales:** 1 adult female (4.0 mm long, 3.2 mm wide):ex open top pit gall in leaf midrib, Eucalyptus sp., Penrith, 24 Nov., 1899, WWF No. 304 (ASCU).

###### Paratypes:

**AUSTRALIA: Victoria:** 3 adult females (1 slide), 6 first-instar nymphs (including NH205), 7 embryos, 1 leaf with vacated pit galls: insects from under bark of Eucalyptus sp. (perhaps Eucalyptus microcarpa), ca 10 km NNW of Benalla, Midland Highway, near turnoff to Hwy C371 to Tocumwal, -36.47°; 145.94°, 29 Dec., 2008, PJG, NH205 (ANIC).

##### Comments.

Adult females of Opisthoscelis tuberculata are closely related to Opisthoscelis thurgoona with which they share well-developed fore, mid and hind legs, 7 distinct antennal segments, a ventral anal opening, with a sclerotic anal ring bearing numerous setae, each of which is surrounded at the base by a number of minute pores. These features of the adult females of Opisthoscelis tuberculata and Opisthoscelis thurgoona are shared with species of Lachnodius. One morphological clue of the closer relationship between Opisthoscelis tuberculata and Opisthoscelis thurgoona and the other Opisthoscelis species (recovered by analysis of DNA sequence data (NBH, unpublished data)) is the presence of macrotubular duct clusters on the dorsum (possibly homologous to the cribriform plates found in Opisthoscelis subrotunda and Opisthoscelis beardsleyi). However, as in Lachnodius, Opisthoscelis tuberculata has 5 fleshy setae on each antenna (4 on Opisthoscelis thurgoona).

The adult female of Opisthoscelis tuberculata can be distinguished readily from that of Opisthoscelis thurgoona as follows (features of Opisthoscelis thurgoona in parentheses): (1) 5 fleshy setae on each antenna (4 on each); (2) dorsal ducts in clusters of up to 30 ducts (2–8 ducts per cluster); and (3) the adult female sits in an open-top leaf pit (in an enclosed leaf gall in Opisthoscelis thurgoona).

The holotype of Opisthoscelis tuberculata is from the collection of W.W. Froggatt and the female was removed from an open pit gall in a leaf midrib and slide-mounted by J.W. Beardsley. Froggatt’s first accession notebook (Gullan 1984b) records collection #304 as “Dactylopius eucalypti.? Penrith (no.2) (Berlese 234). small ascelis like gall”. The reference to Berlese may refer to Professor Antonio Berlese (dal Laboratorio di entomologia agraria, Portici, Italy), because two nearby entries in the notebook say “Sent to Berlese” and give different numbers. The 3 adult females from Victoria were collected dead (post-oviposition), in association with their salmon-pink eggs, under loose bark just above the ground on the main trunk of a stunted tree (about 1.5 m high) growing in the middle of a picnic area car park. Several leaves on the tree had vacated pit galls (Fig. 1k), which are presumed to have been the feeding sites of the females prior to their movement to the bark for oviposition. The live eggs were maintained in a small plastic container and started eclosing on 1 Jan., 2009; DNA was extracted from the first-instar nymphs by NBH.

There is only minor variation between the adult female holotype from New South Wales and the three paratype females from Victoria; in particular, there are fewer clusters of macrotubular ducts on the dorsal submargin of the Victorian collection.

##### Etymology.

The species name is a manuscript name of the late J. W. Beardsley who was describing the species as part of a revision of Lachnodius. We assume that his name refers to the sclerotised dorsal duct clusters.

#### Opisthoscelis ungulifinis

Hardy & Gullan sp. n.

urn:lsid:zoobank.org:act:D355540B-0C13-483A-BA3C-B6189F1BD8D3

Figs 1l 10 

##### Gall

(Fig. 1l).

###### Female.

On leaf. Raised, rather nipple-like excrescences; height ca 2 mm above leaf and projecting 1 mm below, width 3–5 mm. Gall opening star-like, with fissures radiating from central orifice, apparently on adaxial leaf surface.

###### Male.

Unknown.

##### Adult female

**Figure 10.**
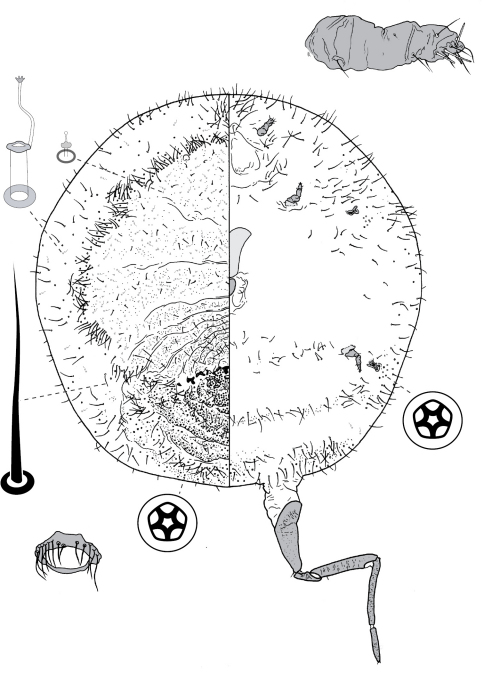
Adult female of Opisthoscelis ungulifinis Hardy & Gullan, sp. n.

(Fig. 10) (n = 7). Body outline circular, with venter broader than dorsum of mature specimens; length 3.0–3.9 mm, greatest width 2.8–3.7 mm; abdomen about as long as head + thorax. Eyespots on dorsum near margin, each 48–58 mm wide. Antennae 3–5 segmented, each 185–230 mm long. Frontal lobes difficult to discern in some specimens, each 330–510 µm long, 185–230 µm wide. Tentorial box 480–530 mm long. Labium 140–155 mm long, 125–175 mm wide. Pump chamber 28–33 µm long, 35–38 µm wide. Spiracles 135–170 mm long, 65–90 mm wide across atrium. Fore and mid legs reduced but segmented, 245–410 µm long. Hind legs slender and elongate; coxa on a fleshy projection, coxa 650–745 µm long, trochanter + femur 820–880 µm long, tibia straight, 645–680 µm long, tarsus 350–410 µm long; claw and digitules present but reduced; translucent pores present on both surfaces of each leg segment except trochanter; each side of trochanter with 3 campaniform sensilla; femur-tibia articulation functional. Anal opening on venter, 58–68 µm wide, anal ring 83–110 µm wide, with 14–20 setae.

###### Dorsum.

Delineated by marginal fringe of elongate (up to 300 µm) flagellate setae, fringe extending from head to abdominal segment IV, margin of each of abdominal segments V–VIII with pair of blunt sclerotic projections at end of short fleshy lamella. Derm densely beset with microtrichia. Dorsal setae flagellate, 15–148 mm long; scattered over dorsum. Macrotubular ducts 14–15 µm long, dermal orifice opening with rim 5 µm wide; ducts in a transverse row across each abdominal segment, scattered over head and thorax. Microtubular ducts 5 µm long, dermal orifice with rim 2 µm wide; distributed evenly over dorsum Quinquelocular pores absent.

###### Venter.

Ventral body surface greatly expanded, posterior abdominal segments only visible from dorsal aspect. Oral lobes membranous. Ventral setae 13–250 mm long, in transverse row across each body segment, scattered along margin. Macrotubular ducts similar to those on dorsum, scattered along margin, in a transverse row across each of abdominal segments II–V. Quinquelocular pores 5 mm in diameter, in a cluster around each spiracle, scattered along margin, in a transverse row across each abdominal segment, most dense around vulva.

**Figure 11.**
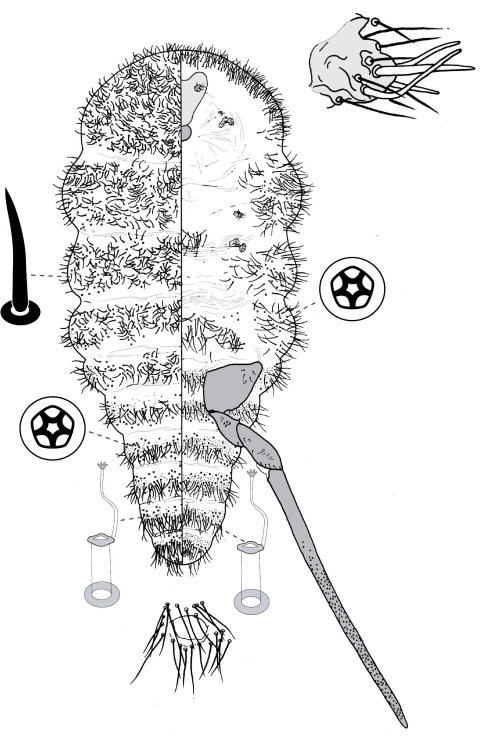
Adult female of Tanyscelis conica (Fuller).

##### Material examined.

###### Holotype of Opisthoscelis ungulifinis (here designated): 

**AUSTRALIA: South Australia:** 1 adult female (3.9 mm long, 3.7 mm wide): ex gall, Eucalyptus ?oleosa, 2 km SW of Oodla Wirra, 5 Dec., 1981, PJG (ANIC).

###### Paratypes:

**AUSTRALIA: South Australia:** 6 adult females, 24 first-instar nymphs: same data as holotype (ANIC).

##### Comments.

Adult females of Opisthoscelis ungulifinis can be recognised easily by the 1 or 2 blunt spines on each of the marginal projections on the posterior abdominal segments, and by having the hind coxae each set on a fleshy projection. Adult females of Opisthoscelis ungulifinis are also unique amongst Opisthoscelis species in having microtubular ducts. The adult females are most similar to those of Opisthoscelis serrata, which also have marginal projections each with 1 or 2 spines; however these spines are conical in Opisthoscelis serrata. Adult females of Opisthoscelis ungulifinis can be further separated from those of Opisthoscelis serrata by lacking marginal projections cephalad of abdominal segment IV (present on all abdominal segments of Opisthoscelis serrata), and by having a marginal fringe of elongate setae (absent in Opisthoscelis serrata).

##### Etymology.

The species name is a combination of the Latin words *ungula*, meaning hoof, and *finis*, meaning end or boundary. It refers to the blunt sclerotic projections at the apex of each marginal lamella. The name is a noun in apposition.

#### Opisthoscelis gracilis

Schrader nomen dubium

Opisthoscelis gracilis Schrader 1863: 7; synonymy with Opisthoscelis subrotunda by Froggatt 1894a: 209.

##### General.

This species was described briefly by Schrader (1863: 7) as follows: “In another species Opisthoscelis gracilis, which I have observed, the oviparous female is rather slender, and the legs are still longer and thinner than in the species before noticed [Opisthoscelis subrotunda], and the male has no anal setae.” There is no illustration and Schrader’s specimens were destroyed in WWII (Gullan 1984a). Froggatt (1894a) considered Opisthoscelis gracilis to be a synonym of Opisthoscelis subrotunda. However, Schrader’s description of the adult female having long thin legs and a slender body, and the adult male with no anal setae, suggest that this was a species of Tanyscelis. In the absence of type specimens, it is impossible to determine which species this name applies to and we here consider it to be a *nomen dubium*.

### Tanyscelis

Hardy & Gullan gen. n.

urn:lsid:zoobank.org:act:07651A23-7D06-488B-8BC9-E59F868031D2

#### Type species:

Opisthoscelis pisiformis Froggatt 1894b.

#### Generic diagnosis.

##### Adult female and associated gall.

Galls on leaves and stems; each either globular, hemispherical, conical, mammiform or thorn-like, never pit-like; with orifice usually slit-like, sometimes circular to oblong; if galls on leaves, orifice on either abaxial or adaxial surface, depending on Tanyscelis species, but surface often difficult to determine in mature isobilateral leaves and galls typically opening on same surface on any one leaf. Female attached by mouthparts to internal gall wall opposite to opening, with long hind legs projecting forwards over body or towards gall orifice; body of female fills only part of gall cavity; in some species dorsum often covered with white powdery wax. Body outline ovate to turbinate. Abdomen tapered. Vulva on ventral surface near apex of abdomen between segments VII and VIII. Eyes on dorsum close to margin or a papilliform fleshy protuberance on each side of head in place of eyes. Antennae reduced, with ≤ 3 segments, usually 1-segmented. Pair of broad frontal lobes posteromedial of antennae (possibly absent in Tanyscelis villosigibba). Oral lobes membranous to sclerotic, sometimes forming large circular pad around mouthparts. Tentorial box with aliform extensions. Fore and midlegs reduced, segmentation indistinct. Hind legs usually positioned on extreme posterior of thorax and often appear to be located on anterior abdomen; elongate, sometimes nearly as long or longer than body; ratio of length of trochanter + femur / length of tibia + tarsus < 1; tibia-femur articulation either functional or tibia and femur fused to varying degrees; translucent pores present at least tibia or tibiotarsus; trochanter with 2–6 campaniform sensilla on each surface; claw always highly reduced to absent, claw and tarsal digitules often present but reduced. Anal opening apical or dorsal; anal ring absent or small, without pores and no more than 6 minute setae. Posterior abdominal segments usually without marginal fleshy projections bearing spines (present near anal opening in Tanyscelis verrucula and Tanyscelis pisiformis). Margin without linear fringe of differentiated setae (though Tanyscelis convexa with submarginal band of staggered spines on venter extending from anterior portion of head to posterior spiracles). Dorsum may be humped, or have large fleshy projections. Derm membranous, sclerotic, or bearing minute papillae. Dorsal setae usually flagellate, minute to very long, 5–220 µm long, sometimes born upon fleshy spines. Cribriform plates and tubular duct clusters absent. Microtubular ducts absent from both body surfaces. Macrotubular ducts with vestibule thin and sclerotic, may be present on dorsum and venter, dorsum only, venter only, or absent. Quinquelocular pores on ventral body surface, sometimes throughout dorsum or confined to dorsal surface of abdomen.

##### Adult male.

Antenna 9-segmented. Abdomen tapered, elongate in some species. Gland pouches and associated setae absent. Detailed description given by Theron (1968). Theron based his description on specimens of two unidentified species collected from Keith, South Australia, ex Eucalyptus fasciculosa, provided to him by HMB. Exactly two species from this host and locality, and collected prior to 1968, are present in the collection of HMB: Opisthoscelis spinosa and Opisthoscelis maculata, here transferred to Tanyscelis.

In contrast, the known adult males of Opisthoscelis have 10-segmented antennae, a non-tappered abdomen, and have gland pouches, each with a pair of gland setae.

##### First-instar nymph.

Anterior margin of head incised at midline. Each spiracle with one trilocular pore next to opening. One submedial longitudinal row of dorsal setae on each side of body. Antennae 3- or 4-segmented, with 4 fleshy setae on apical segment(s). The known first-instar nymphs of Opisthoscelis share all of these states.

#### Etymology.

The genus name is a Latinised combination of the Greek *tany-* meaning long (from the word *tanyo* meaning stretch out) and skelos meaning leg, and refers to the long hind legs of the adult female. Our new name is feminine as for Opisthoscelis, the sister genus of Tanyscelis.

#### Tanyscelis conica

(Fuller) comb. n.

Figs 2a,b 11

Opisthoscelis conica Fuller 1897: 1346; Fuller 1899: 464.

##### General.

Fuller’s (1897: 1346) original description of Opisthoscelis conica is very brief: “Upon one side of the leaf arises the conical apex, whilst on the other the gall protrudes as a hemisphere.” Later Fuller (1899) published a more detailed description with an illustration of three galls of females on a leaf and a hind leg of an adult female (plate XV, figs 33–34). The galls in Fuller’s drawing are very similar to syntype galls housed in the SAMA (see “Material examined” for details), but no insects have been found in the SAMA or elsewhere (e.g., not in the South African National Collection of Insects where some other Fuller material is housed). The type locality of Opisthoscelis conica was given as Midland Junction in Fuller (1897) but as Swan River in Fuller (1899), however it is unlikely that there were two localities for this species because Fuller published the second paper from South Africa based on notes that he had made during eight or nine months spent collecting in Perth prior to his 1897 paper. Midland Junction, now the suburb of Midland, is several km east of the Swan River and, in the 1890s, it was a railway station and associated village.

##### Gall

(Fig. 2a,b).

###### Female.

On leaf. Height 5.0–7.0 mm (= total height above and below leaf blade), width 5.0–9.5 mm. Gall opening slit-like, 0.5–0.8 mm long; on abaxial or adaxial surface, but all galls opening on same surface on any one leaf. Side of gall with opening conical, other side convex, globose (Fig. 2b).

###### Male.

Not known.

##### Adult female

(Fig. 11) (n = 8). Body outline turbinate, margin incised at intersegmental boundaries, length 2.5–3.7 mm, greatest width 1.1–2.5 mm; abdomen tapered, about as long as head + thorax, extending far beyond femur. Eyespots small, each 18–25 mm wide. Antennae 1-segmented, each 35–108 mm long. Frontal lobes difficult to see, each ca 170 µm long, 200 µm wide. Tentorial box 350–470 mm long. Pump chamber 30–43 µm long, 38–48 µm wide. Labium 85–113 mm long, 95–140 mm wide. Spiracles 70–105 mm long, 45–63 mm wide across atrium. Fore and mid legs small sclerotic protuberances; fore leg 20–35 µm long, with 6–8 setae; mid leg larger, 38–80 µm long, with 10–12 setae. Hind legs robust and elongate; coxa 180–260 µm long, trochanter about as a long as femur, the two combined 450–610 µm long, tibia and tarsus fused, forming straight, sword-like segment 720–1880 µm long; claw and digitules absent; translucent pores dense throughout distal region of dorsal and ventral surfaces of tibiotarsus; femur-tibia articulation poorly developed. Anal opening poorly formed, 15–28 µm wide, surrounded by dense cluster of setae.

###### Dorsum.

Derm membranous. Dorsal setae robust, often slightly curved, 15–138 mm long; dense over head and thorax, forming transverse band of longer setae on each abdominal segment. Macrotubular ducts 10–15 mm long, dermal orifice with a rim 5 mm wide; in transverse row on each of abdominal segments III–VI. Microtubular ducts absent. Quinquelocular pores 6–8 µm in diameter, scattered over dorsum.

###### Venter.

Derm on abdomen with bands of microtrichia. Setae 15–150 mm long, in a transverse row across each abdominal segment, dense along margin and submargin of head and thorax, medial cluster on mesothorax and metathorax. Macrotubular ducts similar to those on dorsum, in sparse transverse rows across each of abdominal segments III-VI. Microtubular ducts absent. Quinquelocular pores similar to those on dorsum, similar in distribution to ventral setae.

##### Material examined.

###### Type material:

**AUSTRALIA: Western Australia:** 3 syntype galls of females, dry and empty (2 dissected open, third gall parasitised), on 2 leaves, all on pin-mount, 2 labels: “Opisthoscelis Schrader / conica, Fuller, sp. n. Type / Perth, W. Australia / C. Fuller / 28.7.97” and “ Perth, W. A. / Opisthoscelis / conica Fuller / m/s / 28.7.97” (SAMA).

###### Additional material:

**AUSTRALIA: South Australia:** 4 first-instar nymphs: ex galls on leaves, Eucalyptus dumosa, Danggali Conservation Park, Main Road site 1, -33.29°; 140.59°, 21 Apr., 1996, PJG (ANIC); 2 adult females, 35 first-instar nymphs: ex leaf galls, Eucalyptus dumosa, Danggali Conservation Park, Main Road, Site 1, -33.29°; 140.59°, 22 Apr., 1996, PJG (ANIC); 2 adult females: ex leaf galls, Eucalyptus dumosa, Danggali Conservation Park, Main Road, Site 4, -33.28°; 140.59°, 23 Apr., 1996, PJG (ANIC); 2 adult females: ex leaf galls, Eucalyptus dumosa, Danggali Conservation Park, Tipperary Dam– Canopus Road, Site 6, -33.27°; 140.72°, 24 Apr., 1996, PJG (ANIC). **Victoria:** 2 adult females: ex leaf galls, Eucalyptus sp.(mallee), 25 km N of Ouyen, on Calder Hwy, 22 Apr., 1994, T. Murphy (ANIC); 2 adult females: Eucalyptus incrassata, Big Desert, Moonlight Tank, 13 Feb., 1977, PJG (ANIC); 1 adult female: ex leaf gall Eucalyptus incrassata, Big Desert, Wyperfeld National Park, 13–18 Aug., 1977, T. P. O’Brien (ANIC); 2 adult females: ex leaf galls, Eucalyptus dumosa, ca 20 km N of Hattah, Calder Highway, -34.68°; 142.25°, 5 Feb., 2005, PJG and NBH, NH48 (ANIC); 1 adult female: ex leaf gall, Eucalyptus dumosa, ca 20 km W of Mittyack, Calder Highway, -35.17°; 142.45°, 5 Feb., 2005, PJG and NBH, NH76 (ANIC). **Western Australia:** 6 adult females: ex leaf galls, Eucalyptus ?wandoo, 2.3 km S of Boddington and 18 km SSW of North Bannister, 5 Jan., 1986, PJG (ANIC); 2 adult females: ex leaf galls, Eucalyptus wandoo, 44 km N of Williams, Albany Hwy, 29 Mar, 1978, PJG (ANIC); 3 adult females, 1 second-instar female: ex leaf galls, Eucalyptus accedens, Coomallo, off Brand Hwy, on dam edge, -30.23°; 115.40°, 3 Dec., 1990, PJG (ANIC); 1 adult female, 3 second-instar females: ex galls on leaves, Eucalyptus wandoo, Woodanilling, Great Southern Hwy, at crossroads, -33.57°; 117.43°, 1 Dec., 1994, PJG (ANIC).

##### Comments.

The adult female of Tanyscelis conica would be difficult to confuse with the adult female of any other known species of Tanyscelis. The fusion of the hind tibia and tarsus into a broad, sword-like appendage is unique. Both the form of the dorsal setae (robust and slightly curved) and their dense distribution also separate adult females of Tanyscelis conica from those of other species. Known populations of Tanyscelis conica occur in two disjunct clusters, one centered around Perth in southwest Western Australia, and the other in forest reserves in northwestern Victoria and southeastern Australia (e.g., Danggali Conservation Park, Wyperfield National Park, Hattah-Kulkyne National Park). Despite the great distance separating the two, ca 2000 km, the morphology is homogeneous. Tanyscelis conica is the only species of Tanyscelis known from Western Australia. Only one species of Opisthoscelis, the type species Opisthoscelis subrotunda, has been collected from Western Australia, in the Kimberley region, which is a great distance (> 1,500 km) north of Perth. Tanyscelis conica is also the only known species of Tanyscelis or Opisthoscelis known to feed on mallee eucalypt species in the section Dumaria, and along with Opisthoscelis ungulifinis, is one of only two species in this group known to feed on eucalypts in the section Bisectae.

#### Tanyscelis convexa

(Froggatt) comb. n.

Figs 2c,d 12

Opisthoscelis convexa Froggatt 1929: 376.
Opisthoscelis globosa Froggatt 1929: 376 (recognised as a junior primary homonym of Opisthoscelis globosaRübsaamen 1894, by Lindinger 1943: 223), syn. n.Opisthoscelis rubsaameniLindinger 1943: 223; replacement name for Opisthoscelis globosa Froggatt.
Opisthoscelis ruebsaameni  Lindinger; justified emendation by Miller and Gimpel 2000: 414.

##### General.

We have examined all available type specimens of Froggatt’s Opisthoscelis convexa and Opisthoscelis globosa (the latter now Opisthoscelis ruebsaameni) (see under “Material examined” for details of types) and inasmuch as the poor quality of the specimens allows comparison, we consider these adult females to be identical. Froggatt (1929) described these two species on the same page of his paper, with Opisthoscelis convexa described first, and based his new species on specimens of each from both Victoria (Diamond Creek for Opisthoscelis convexa but not specified for Opisthoscelis globosa Froggatt) and from New South Wales (Gosford, Macksville and Warrah for Opisthoscelis convexa and Hornsby for Opisthoscelis globosa Froggatt). He stated that the gall of the female of his Opisthoscelis globosa was larger and more swollen, and the adult female was broader with more irregular transverse bands of spines on the dorsal abdominal segments than on the female of Opisthoscelis convexa. We did not observe a striking difference in the dorsal abdominal spines beween Froggatt’s specimens of Opisthoscelis globosa and Opisthoscelis convexa. The size differences he mentions probably relate to the age of the females.

##### Gall

(Fig. 2c,d).

###### Female

(Fig. 2c). On stem and base of petiole. Height 3.0–7.7 mm, width 4.5–8.6 mm, length of basal attachment 4.6–11.8 mm. Gall opening slit-like to oblong, 0.1–0.4 mm wide, 0.4–1.7 mm long (paralectotype galls with slit-like opening mostly 0.1 x 1.2 mm). Gall globose, surface variable, distal area frequently with 1 or 2 concentric circular scars, distal surface above scars smooth and lighter in colour than surrounding stem tissue.

###### Male

(Fig. 2d). On either leaf surface, occasionally on petiole or stems. Height 1.4–3.7 mm, width 1.0–1.6 mm, length of basal attachment 1.3–2.6 mm. Gall cylindrical to conical, distal margin dentate, opening slit-like to round, 0.5–1.7 mm wide, opposite side of leaf swollen.

##### Adult female

**Figure 12.**
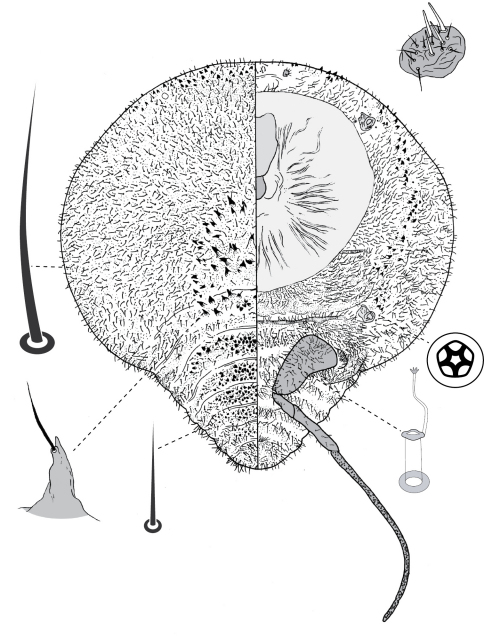
Adult female of Tanyscelis convexa (Froggatt).

(Fig. 12) (n = 24). Body outline broadly turbinate, outline of head + thorax + abdominal segment I entire, abdominal margin weakly incised between each segment, length 2.3–4.4 mm, greatest width 1.9–5.0 mm; abdomen tapered, about as long as head + thorax. Eyespots each 25–83 mm wide, on dorsum among or medial to marginal spines. Antennae 1-segmented, in form of low convex plate or knob; width 25–78 mm. Frontal lobes difficult to see, each ca 220 µm long, 230 µm wide. Tentorial box 350–600 mm long. Pump chamber 50–63 µm long, 45–55 µm wide. Labium 73–160 mm long, 80–125 mm wide. Spiracles 100–205 mm long, 48–118 mm wide across atrium. Fore and mid legs small sclerotic protuberances, 20–45 µm long. Hind legs slender and elongate; coxa 360–670 µm long, hirsute, trochanter + femur 480–880 µm long, tibia 1000–1610 µm long, tarsus 75–250 µm long; translucent pores dense on both dorsal and ventral surfaces of tibia and tarsus; femur-tibia articulation non-functional, tibia fixed in orientation parallel to long axis of femur; claw and digitules absent. Anal opening poorly formed, 8–20 µm wide; sclerotic anal ring and anal ring setae absent.

###### Dorsum.

Derm variously sclerotic, beset with small papillae; medial portions of thorax and abdomen with numerous spines each born on a fleshy protuberance, each spine with ante-apical seta, spines diminishing in size caudad. Dorsal setae flagellate, 20–180 mm long; scattered over dorsum. Macrotubular ducts absent. Microtubular ducts absent. Quinquelocular pores absent.

###### Venter.

Derm with microtrichia on abdominal segments. Oral lobes sclerotic, forming large circular feeding pad. Spines similar to those on dorsomedial areas of thorax and abdomen found in submarginal row extending from anterior margin of head to posterior spiracles. Ventral body surface extremely hirsute, setae similar to those on dorsum, flagellate, 30–160 mm long. Macrotubular ducts 12–15 µm long, dermal orifice with rim 5 µm wide; on medial areas of anterior abdominal segments only. Quinquelocular pores large, 7–10 mm in diameter; small cluster around each spiracle.

##### Material examined.

###### Lectotype of Opisthoscelis convexa Froggatt (here designated):

**AUSTRALIA: Victoria:** 1 adult female on WWF original slide-mount with another female, lectotype (3.4 mm long, 4.0 mm wide) closest to middle of slide, with labels: “Opisthoscelis / convexa / n sp / Eucalyptus / NSW & Vic.” and “O. convexa” (ANIC).

###### Paralectotypes of Opisthoscelis convexa:

**AUSTRALIA: Victoria:** 1 adult female on same slide-mount as lectotype: same data as lectotype (ANIC); 1 adult female, WWF original slide-mount labelled: “Opisthoscelis / convexa / n sp / N. S. Wales / WWF” and “Finished O. convexa” (ANIC); 2 adult females plus associated dry galls on stem, 6 slides of embryos and first-instar nymphs (several hundred) and 7 other dry galls of females: ex Eucalyptus macrorrhyncha, Diamond Creek, Oct., 1921, C. French, 1055, ASCT00004874 and ASCT00004875 (ASCU).

###### Lectotype of Opisthoscelis globosa Froggatt (here designated):

**AUSTRALIA: New South Wales:** 1 adult female (3.8 mm long, 4.0 mm wide): WWF original slide-mount labelled: “Opisthoscelis / globosa / n sp / N. S. Wales / WWF” and “O. globosa” (ANIC).

###### Paralectotype of Opisthoscelis globosa:

1 adult female: WWF original slide-mount labelled: “Opisthoscelis / globosa / n sp” and “O. globosa” (ANIC).

In the BMNH, there are also two collections of dry galls labelled as “Opisthoscelis convexa n. sp.” and apparently from WWF (one from Dandenong, Victoria, and one from Sydney, N.S.W.). These appear to have been sent to London in 1922, prior to Froggatt’s description of this species. In 1985, PJG dissected dry galls from the Dandenong collection and slide-mounted two adult females and some first-instar nymphs (listed below). Although WWF presumably identified these as belonging to his new species, with the manuscript name Opisthoscelis convexa, the collection data for these specimens are not listed in Froggatt’s (1929) description of the species and thus we do not consider them to be part of the type material.

###### Additional material:

**AUSTRALIA: Australian Capital Territory:** 6 adult females: ex galls, Eucalyptus ?melliodora, Canberra, beside road to Red Hill Lookout, 20 June, 1981, D. P. Faith and PJG (ANIC). **New South Wales:** 2 adult females: ex stem galls, Eucalyptus largiflorens, 20 km NE of Swan Hill, Merran Creek, 16 Jan., 1986, PJG (ANIC); 2 adult females: ex stem galls, Eucalyptus ?melliodora, ca 30 km E of Goulburn, ca 2 km E of Bungonia Lookdown, above Bungonia Creek and Shoalhaven River, 5 Oct., 1985, PJG (ANIC); 2 adult females: ex stem galls, Eucalyptus haemastoma, Sydney, 17 Dec., 1921, WWF, #1922-55 (BMNH). **Queensland:** 2 adult females: ex galls, Eucalyptus sp., 20 km NW of Karara, Millmerran Road, -28.08°; 151.45°, 13 May, 1995, LGC (ANIC); 2 adult females: ex galls, Eucalyptus sp., 35 km W of Millmerran, -27.70°; 151.02°, 1 May, 1995, LGC (ANIC); 1 adult female, 5 first-instar nymphs: ex galls, Eucalyptus sp., 45 km SW of Mt Garnet, Kennedy Hwy, -16.97°; 144.86°, 17 Oct., 2003, LGC and M. D. Crisp, LGC00043 (ANIC); 1 adult female: ex gall on stem, Eucalyptus sp., 8 km NW of Karara, Millmerran Road, -28.17°; 151.55°, 13 May, 1995, G. Harper (ANIC); 3 adult females: ex stem galls, Eucalyptus sp., 9 km E of Felton, -27.85°; 151.82°, 1 May, 1995, LGC (ANIC); 1 adult female: ex gall, Eucalyptus cambageana, ca 31 km W of Moura, Dawson Hwy, -24.65°; 149.69°, 4 Nov., 2003, LGC and M. D. Crisp, LGC00076 (ANIC); 4 adult females, 200 first-instar nymphs: ex galls, Eucalyptus melanophloia, ca 50 km W of Townsville, Hervey Range, -19.33°; 146.40°, 2 Sep., 1996, C. A. M. Reid (ANIC); 100 first-instar nymphs: ex galls, Eucalyptus melanophloia, ca 50 km W of Townsville, Hervey Range, 17 Sep., 1996, C. A. M. Reid (ANIC); 1 adult female: ex stem gall, Eucalyptus melliodora, E of Inglewood, 6 km W of Gore on Cunningham Hwy, -28.35°; 151.35°, 15 Aug., 2004, NBH and PJG, NH22 (ANIC); 1 adult female: ex Eucalyptus populifolia [Eucalyptus tereticornis], Barakula, 5 Oct., 1939, INSECOLL 0-067179 (QDPI); 1 adult female: ex Eucalyptus crebra, Blair, Athol, Nov., 1938, INSECOLL 0-067180 (QDPI); 2 adult females: ex Eucalyptus hemiphloia, Brisbane, INSECOLL 0-067174, 0-067175 (QDPI); 5 adult females: ex Eucalyptus crebra, Forest Hill, 12 Sep., 1938, No. 339, ISECOLL 0-067177, 0-067178, 0-067187 (QDPI); 1 adult female: ex Eucalyptus hemiphloia, Gallangowan, 15 Feb., 1944, No. SC1953, INSECOLL 0-067183 (QDPI); 2 adult females: ex stem galls, Eucalyptus sp., Great Keppel Island, Lighthouse track, -23.17°; 150.97°, 14 Dec., 1993, LGC (ANIC); 1 adult female: Eucalyptus sp. (sapling), intersection of Boomerang Road and Beenleigh–Beaudesert Road, -27.78°; 153.20°, 2 May, 1993, PJG (ANIC); 2 adult females: ex Eucalyptus drepanophylla, Kandanga, 13 Apr., 1961, INSECOLL 0-067185 (QDPI); 1 adult female: ex gall, Eucalyptus siderophloia, Miriam Vale to Monto Road, Blackman Gap, 351 m, -24.44°; 151.44°, 5 Nov., 2003, LGC and M. D. Crisp, LGC00080 (ANIC); 1 adult female: ex gall, Eucalyptus pilligaensis, nr Koorongara, -28.03°; 151.27°, 2 May, 1995, L. G. Cook (ANIC); 2 adult females: ex Eucalyptus hemiphloia, R8 Doongul, 25 Jul., 1939, No. SC777, INSECOLL 0-067181, 0-067182 (QDPI); 2 adult females: ex Eucalyptus crebra, Western Line, 3 Oct., 1938, INSECOLL 0-067188 (QDPI); 1 adult female: ex Eucalyptus crebra, no locality data, 23 Dec., 1937, INSECOLL 0-067184 (QDPI); 10 adult females: no data, INSECOLL 0-067169 – 0-067172. **South Australia:** 8 adult females, 55 first-instar nymphs: ex stem galls, Eucalyptus fasciculosa, Belair, National Park, 25 Nov., 1963, HMB, Specimen Index No. 50/63 (ANIC); 10 adult female: ex galls, Eucalyptus sp., E of Victor Harbor, 9 Oct., 1967, HMB, Specimen Index No. 32/67 (ANIC); 1 adult female: Eucalyptus sp., Mt Bold Reservoir, 18 Sep., 1966, N. L. Berlinsky, Specimen Index No. 38/66 (ANIC). **Victoria:** 3 adult females: ex stem galls, Eucalyptus microcarpa, 8 km W of Melton, 27 Feb., 1975, PJG (ANIC); 1 adult female: ex stem gall, Eucalyptus albens, ca 10 km NNW of Benalla, Casey’s Weir, Broken River, -36.48°; 145.95°, 8 Feb., 2004, PJG, LGC00104 (ANIC); 1 second-instar female: ex stem gall, Eucalyptus microcarpa, ca 10 km S of Nagambie, on road to Avenel, near railway line, -36.38°; 145.17°, 30 Jan., 2005, PJG, NH27 (ANIC); 2 adult females: ex stem galls, Eucalyptus polyanthemos, Melbourne, North Warrandyte, corner of Overbank Road and Glynns Road, -37.73°; 145.20°, 14 Feb., 2005, PJG and NBH, NH28 (ANIC); 3 adult females: Eucalyptus microcarpa, nr Bacchus Marsh, Long Forest Road, 19 Jan., 1976, PJG (ANIC); 1 adult female: ex gall, Eucalyptus ?microcarpa, Shepparton, near Goulburn River, off Tom Collins Drive, -36.39°; 145.39°, 29 Dec., 2004, PJG, NH55 (ANIC); 2 adult females, 2 slides with first-instar nymphs: ex stem galls, Eucalyptus elaeophora [now Eucalyptus goniocalyx], Dandenong, WWF, 17 Dec. 1921, 1922/55 (BMNH).

##### Comments.

Adult females of Tanyscelis convexa can be recognised easily by the large dorsal spines found on the dorsomedial areas of the thorax and abdomen and in a ventral submarginal row extending from the anterior margin of the head to the posterior spiracles. Tanyscelis convexa is also the only species of Tanyscelis to have no tubular ducts or multilocular pores on the dorsum. DNA sequence data support a sister relationship between Tanyscelis convexa and Tanyscelis maculata + Tanyscelis maskelli (NBH, unpublished data). Tanyscelis convexa is one of the more frequently collected, and broadly distributed, species of Tanyscelis. It is known to occur along the coast from Adelaide in South Australia to Cairns in north Queensland. Adult females from near Swan Hill but in New South Wales, and from Canberra differ from all other collections of Tanyscelis convexa in having the ventromarginal band of spines poorly developed. Samples from Benalla, Shepparton, and Nagambie (more or less between but about 100 km farther south than the Canberra and Swan Hill samples) have the ventromarginal band of spines well developed. Tanyscelis convexa has been collected exclusively from eucalypt species in the section Adnataria.

#### Tanyscelis grallator

Hardy & Gullan sp. n.

urn:lsid:zoobank.org:act:F524C2C7-194B-43D4-A10B-D8A716C8D083

Fig. 2e 13 

##### Gall

(Fig. 2e).

###### Female.

On leaf. Thorn-like, height 2.2–3.0 mm, width 3.0–4.8 mm, length of basal attachment 5.0–6.0 mm. Gall opening slit-like, 0.5–0.8 mm long; on adaxial leaf surface for several galls examined. Base broad and globose with circular scar; apex tapering to blunt point. Live adult females of Tanyscelis grallator have white powdery wax in a band across dorsum of each body segment, with intersegmental areas bare of wax. The female is small in relation to the size of the gall cavity and eggs are laid into the cavity, which can become filled.

###### Male.

Not known.

##### Adult female

**Figure 13.**
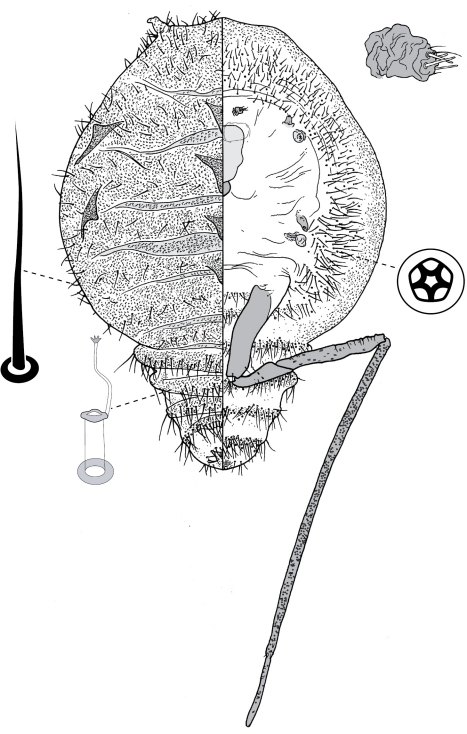
Adult female of Tanyscelis grallator Hardy & Gullan, sp. n.

(Fig. 13) (n = 10). Body turbinate, head, thorax, and anterior abdominal segments with intersegmental boundaries indistinct along margin, intersegmental boundaries incised along posterior abdominal margin; body length 2.4–2.7 mm, greatest width 1.7–2.0 mm; abdomen tapered, about as long as head + thorax, not extending beyond outstretched femur. Large (115–215 mm long), papilliform fleshy protuberance on each side of head in place of eyes; apex of each protuberance with differentiated area, sometimes surrounded by invagination. Antennae each 1-segmented, 55–88 mm long. Frontal lobes difficult to see, each 125–255 µm long, 145–245 µm wide. Tentorial box 310–410 mm long. Labium 88–113 mm long, 68–93 mm wide. Pump chamber 30–38 µm long, 30–38 µm wide. Spiracles 83–128 mm long, 53–73 mm wide across atrium. Fore leg 43–88 µm long; mid leg 68–163 µm long. Hind leg slender and elongate; coxa cylindrical, 520–630 µm long, trochanter + femur 770–960 µm long, tibia 1200–1900 µm long, tarsus 255–420 µm long; translucent pores dense on both surfaces of tibia, few on tarsus; trochanter with 2 campaniform sensilla on each side; femur-tibia articulation functional; claw and digitules present but reduced. Anal opening 10–20 µm wide, without distinct sclerotic anal ring.

###### Dorsum.

Derm weakly sclerotised, densely beset with small papillae; a conical to papilliform protuberance medially on each thoracic segment and on abdominal segment I, and a similar protuberance on each submargin of thoracic segments I and II, smaller protuberances may be present on each submargin of anterior abdominal segments. Dorsal setae flagellate, 25–212 mm long; arranged in a transverse row across each abdominal segment, scattered over surface of head and thorax; setae increasing in length caudad. Macrotubular ducts 6–12 mm long, dermal orifice with rim 4–5 mm wide; in transverse row across each abdominal segment, scattered over head and thorax. Microtubular ducts absent. Quinquelocular pores absent.

###### Venter.

Derm with microtrichia on abdomen. Oral lobes membranous. Setae 68–200 mm long, in a transverse row across each abdominal segment, along margin of head and thorax. Macrotubular ducts absent. Quinquelocular pores large, 8–10 µm in diameter, similar in distribution to ventral setae.

##### Material examined.

###### Holotype (here designated):

**AUSTRALIA: Queensland:** 1 adult female (2.4 mm long, 2.0 mm wide): ex conical gall on leaf, Eucalyptus sp., 29 km N of Normanton, -17.47°; 141.18°, 1 Oct., 2006, LGC, LGC00666 (ANIC).

###### Paratypes:

**AUSTRALIA: Queensland:** 2 adult females: ex leaf gall, Eucalyptus sp. (ironbark), 13 km N of Injune, 7 Dec., 1993, LGC (ANIC); 1 adult female: ex Eucalyptus sp., Charters Towers, Oct., 1955, ARB, INSECOLL 0-067153 (QDPI); 1 adult female: ex Eucalyptus crebra, Clermont, Nov., 1938,INSECOLL 0-067159 (QDPI); 3 adult females: ex leaf galls, Eucalyptus sp. (ironbark), Dunmore State Forest, 2 May, 1995, G. Harper (ANIC); 4 adult females: ex leaves, Eucalyptus crebra, Emu Vale, 15 Mar., 1939, INSECOLL 0-067161, 0-067162 (QDPI); 1 adult female: ex gall, Eucalyptus crebra, Mt Walsh Nat. Park, picnic ground, 267 m, -25.57°; 152.05°, 11 Oct., 2003, LGC and M. D. Crisp, LGC00029 (ANIC); 1 adult female: no data, INSECOLL 0-067168 (QDPI).

##### Comments.

Adult females of Tanyscelis grallator are most similar to those of Tanyscelis mollicornuta. Most distinctively, adult females of both species have fleshy protuberances in place of eyes. These protuberances are much longer on Tanyscelis mollicornuta (≥ length of hind tarsus) than on Tanyscelis grallator (<< length of hind tarsus), which has additional protuberances along the dorsal midline plus the submargin of the thorax. Each protuberance on the head of Tanyscelis grallator has the apex much more differentiated than the similar protuberances of Tanyscelis mollicornuta, and it seems possible that this area is light sensitive. Adult females of Tanyscelis grallator can be distinguished further from those of Tanyscelis mollicornuta by having much longer hind legs, each with a cylindrical coxa (more conical in Tanyscelis mollicornuta), and the ratio of the hind trochanter + femur to tibia + tarsus << 1 (ca 1 in Tanyscelis mollicornuta). Tanyscelis mollicornuta is known from only one location, in Queensland, east of Western Creek State Forest, near Milmerran. Samples of Tanyscelis grallator have been collected from five locations in Queensland, including a collection from the eastern edge of Kumbarilla State Forest, < 50 km to the north of Milmerran. Tanyscelis mollicornuta and Tanyscelis grallator have only been found on eucalypts in the subgenus Adnataria.

##### Etymology.

The species name is derived from the Latin word *grallator*, meaning ‘stilt walker’, in reference to the extremely long, slender legs of the adult female. The name is a noun in apposition.

#### Tanyscelis maculata

(Froggatt) comb. n.

Figs 2f,g 14

Opisthoscelis maculata Froggatt 1894b: 345–346.
Opisthoscelis recurva Froggatt 1929: 377, syn. n.

##### General.

We have synonymised Opisthoscelis recurva with Opisthoscelis maculata. We could find no morphological differences between the adult females of each, although type adult females of Opisthoscelis recurva tend to be larger than those of Opisthoscelis maculata. Furthermore, the ‘recurved’ gall morphology from which the name of the junior synonym was derived is found occasionally in galls of smaller specimens that match the types of Opisthoscelis maculata. The type locality for Opisthoscelis maculata is Bendigo, Victoria, with galls reported from Eucalyptus leucoxylon and Eucalyptus gracilis (Froggatt 1894b), whereas Opisthoscelis recurva was described from specimens collected on the hills at Warrah Station, a farming property near Willow Tree [not Willowtree as in the description], New South Wales, on an undetermined species of Eucalyptus (Froggatt 1929).

##### Gall

(Fig. 2f, g).

###### Female.

On stem (mostly), fruit, leaf petiole and midrib. Conical (Fig. 2f) to thorn-like (Fig. 2g), height 3.2–16.2 mm, width 2.7–6.9 mm, length of basal attachment 3.6–11.2 mm. Gall opening oblong, 0.1–0.4 mm wide, 0.3–1.2 mm long. Base of gall broad, surface smooth, distal portion attenuated and annulated, may be recurved, 1–6 rings.

###### Male.

On stem and either leaf surface (Fig. 2f), height 1.9–8.0 mm, width 0.9–2.8 mm, length of basal attachment 1.1–3.8 mm. Gall thin-walled and cylindrical, distal edge crenulate, opening round to oblong, 0.3–2.4 mm maximum wide.

##### Adult female

**Figure 14.**
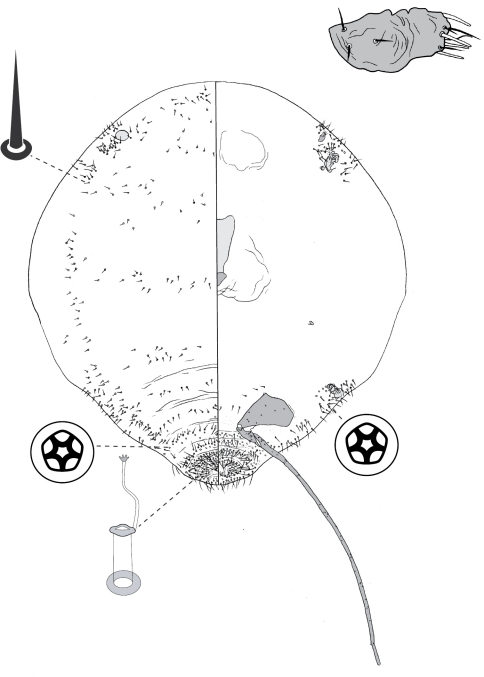
Adult female of Tanyscelis maculata (Froggatt).

(Fig. 14) (n = 53). Body outline ovate to turbinate, length 1.7–5.6 mm, greatest width 0.9–4.6 mm; abdomen tapered, quite small relative to thorax and head. Eyespots large, each 45–123 mm wide, highly convex, surrounded by dense cluster of stout setae. Antennae each 1-segmented, 45–520 mm long. Frontal lobes difficult to see, each 180–650 µm long, 180–580 µm wide. Tentorial box 260–520 mm long. Pump chamber 33–60 µm long, 34–55 µm wide. Labium 50–163 mm long, 60–138 mm wide. Spiracles 85–240 mm long, 38–130 mm wide across atrium. Fore and mid legs small sclerotic protuberances, 15–120 µm long. Hind legs slender and elongate; coxa 240–730 µm long, trochanter + femur 240–930 µm long, tibia 840–4500 µm long, tarsus 140–350 µm long; translucent pores sparsely scattered on dorsal and ventral surfaces of tibiotarsus; trochanter with 3 or 4 campaniform sensilla on each side; femur-tibia articulation non-functional, tibia fixed in orientation parallel to long axis of femur; claw and digitules absent. Anal opening poorly formed, 10–45 µm wide, sclerotic anal ring and anal ring setae absent.

###### Dorsum.

Derm membranous in young females, older specimens may have clusters of small circular sclerotic regions on margin and submargin of head and thorax, or broad areas of sclerotic cuticle on medial portions of head and thorax. Dorsal setae robust, 10–125 mm long; arranged in a transverse band across each body segment and scattered along margin, longer setae present on abdominal segments. Macrotubular ducts 12 mm long, dermal orifice with rim 5 mm wide; restricted to posterior abdominal segments. Microtubular ducts absent. Quinquelocular pores, 7–11 µm in diameter, scattered across posterior abdominal segments.

###### Venter.

Setae 20–165 mm long, in a transverse row across each posterior abdominal segment plus a cluster extending from each spiracle to margin, a few mesad to each metacoxa, otherwise ventral body surface naked. Macrotubular ducts absent. Quinquelocular pores large, 7–11 mm in diameter, similar in distribution to ventral setae.

##### Material examined.

###### Lectotype of Opisthoscelis maculata (here designated):

**AUSTRALIA: Victoria:** 1 adult female (2.3 mm long, 2.2 mm wide): ex dry gall on stem, with printed label: “No. 1787 E / GALL MAKING COCCIDS. / Opisthoscelis maculata, Frogtt. / Female galls on Eucalyptus / Bendigo, Victoria“, ASCT00004880 (ASCU).

###### Paralectotypes of Opisthoscelis maculata:

**AUSTRALIA: Victoria:** 1 adult female, same data as lectotype (ASCU).

###### Lectotype of Opisthoscelis recurva (here designated):

**AUSTRALIA: New South Wales:** 1 adult female (5.2 mm long, 4.1 mm wide): on WWF original slide-mount labelled: “Opisthoscelis / recurva / n sp / Warrah NSW” (ANIC).

###### Paralectotypes of Opisthoscelis recurva:

**AUSTRALIA: New South Wales:** 1 adult female and 2 dry galls of female on base of leaves (female ex one of these galls slide-mounted by PJG 2008), with WWF labels: “Warrah / Sydney, N.S.W. / W. W. Froggatt. / Eucalypt / 8.5.1921” and “1045”, ASCT00004859 & ASCT00004860 (ASCU); 1 adult female and 2 slides of first-instar nymphs ex dry gall on leaf, plus other associated dry galls, with 2 labels: “1579 / Opisthoscelis / recurva / n sp MS / WWF Warrah, N.S.W.” and “Opisth. recurva. (Frogg. MS) / on Eucalyptus sp. / Warrah. N.S.W., Australia” (BMNH). Note: The labels on the 2 dry type galls of Opisthoscelis recurva at ASCU say “Warrah / Sydney” but this is an error; Warrah is property near Willow Tree, in northeastern N.S.W. The paralectotypes in the BMNH are labelled correctly.

###### Additional material:

**AUSTRALIA: New South Wales:** 1 adult female: ex dry stem gall, Eucalyptus sp., WWF, 1919-299 (BMNH); 10 adult female: ex gall on stem, Eucalyptus melliodora, 1 km N of Thurgoona (Albury), -36.05°; 146.98°, 30 Dec., 1991, PJG (ANIC); 1 adult female: ex stem gall, Eucalyptus polyanthemos, 2 km NE of Thurgoona, Ettamogah Rd, -36.03°; 146.98°, 8 Feb., 2004, PJG, LGC00134 (ANIC); 1 adult female: ex gall, Eucalyptus camaldulensis, Deniliquin Landcare Project, 10 June, 1993, P. Bacon, Tree 2-1-5-N (ANIC); 1 adult female: ex dry stem gall, Eucalyptus sp., [locality not given], WWF, #1919-299” (BMNH). **Queensland:** 3 adult females: ex stem galls, Eucalyptus populnea, 3 km WSW of Millmerran, Turallin Rd, -27.88°; 151.24°, 1 May, 1995, LGC (ANIC). **South Australia:** 2 adult females: ex conical twig galls, Eucalyptus fasciculosa, Aldinga Beach, scrub area, 25 Apr., 1972, J. W. Beardsley (ANIC); 5 adult females: Eucalyptus sp., Hindmarsh Waterfall, 10 Oct, 1966, N. L. Berlinsky (ANIC); 5 adult females, 140 first-instar nymphs: ex stem and leaf galls, Eucalyptus lansdowneana subsp. albopurpurea, Kangaroo Island, South Coast Rd, at Eleanor River, -35.93°; 137.23°, 29 Sep., 1994, PJG (ANIC); 27 adult females: ex galls, Eucalyptus fasciculosa, Keith, 18 Feb., 1963, T. C. R. White, Specimen Index No. 16/63 (ANIC); 12 second-instar females: Eucalyptus fasciculosa, Keith, Dec., 1963, T. C. R. White, Specimen Index No. 63/63 (ANIC); 6 adult females: ex galls, Eucalyptus fasciculosa, Keith, 9 Jan., 1964, T. C. R. White (ANIC); 4 adult females: ex galls, Eucalyptus sp., Mt Bold area, 6 May, 1968, V. Cruickshank (ANIC); 3 adult females: Eucalyptus sp., Mt Bold Reservoir, 18 Sep., 1966, N. L. Berlinsky (ANIC); 5 adult females: ex galls, Eucalyptus fasciculosa, Mt Lofty Ranges, Kuitpo Forest Reserve, 13 Mar., 1977, R. C. Robinson and PJG (ANIC); 22 adult females: ex galls, Eucalyptus fasciculosa, Mt. Lofty Ranges, Kuitpo Forest Reserve, 13 Mar., 1977, PJG (ANIC); 12 adult females, 18 first-instar nymphs: ex galls, Eucalyptus sp., nr Willunga, 7 July, 1965, HMB, Specimen Index No. 25/65 (ANIC); 3 adult females, 55 first-instar nymphs: Eucalyptus fasciculosa, Tintinara [near Keith], 4 July, 1963, T. C. R. White, Specimen Index No. 34/63 (ANIC); 11 adult females: ex galls, Eucalyptus fasciculosa, Wilpena Pound, Pound Gap, 24 May, 1977, PJG (ANIC). **Victoria:** 1 adult female: ex stem gall, Eucalyptus ?melliodora, 9 km N of Nagambie, Weir Road, -36.72°; 145.18°, 7 Feb., 2004, PJG, LGC00137 (ANIC); 5 adult females including DNA voucher LGC00103: ex stem gall, Eucalyptus ?microcarpa, ca 10 km S of Nagambie, just E of railway line, -36.37°; 145.17°, 7 Feb., 2004, PJG (ANIC); 2 adult females, 1 second-instar female: ex galls on stems and leaf bases, Eucalyptus polybractea, ca 3 km SW of Inglewood, on Arnold West–Inglewood Road, ca 1 km from Inglewood– Rheola Road, -36.59°; 143.85°, 3 Feb., 2005, PJG and NBH, NH74 (ANIC, NMV); 2 adult females: ex stem galls, Eucalyptus melliodora, ca 9 km N of Nagambie, Goulburn Weir Road, -36.72°; 145.18°, 30 Jan., 2005, PJG, NH25 (ANIC); 5 adult females: ex small, conical galls on twigs, Eucalyptus melliodora, Melbourne, Lower Plenty, 16 Oct., 1971, J. W. Beardsley, V-127 (BPBM); 2 adult females: ex twig galls, Eucalyptus melliodora, Melbourne, Lower Plenty, 1 Jan., 1972, J. W. Beardsley (BPBM); 90 first-instar nymphs: Eucalyptus melliodora, Melbourne, Park Orchards, 18 & 30 Oct., 1976, J. Nelson (ANIC); 4 adult males: Eucalyptus melliodora, Melbourne, Park Orchards, 4 & 7 Jan., 1977, J. Nelson (ANIC); 7 adult females: Eucalyptus melliodora, Melbourne, Park Orchards, 27 Feb., 1976, J. Nelson (ANIC); 2 adult females: Eucalyptus polyanthemos, Melbourne, Warrandyte, behind 134 Brackenbury Street, 7 May, 1977, PJG (ANIC); 2 adult females: Eucalyptus macrorrhyncha, nr Heathcote, 24 Feb., 1972, J. W. Beardsley (ANIC); 1 adult female: ex twig gall, Eucalyptus sp., Wattle Park (reserve), nr Melbourne, 8 Feb., 1964, HMB, Specimen Index No. 10/64 (ANIC).

##### Comments.

Adult females of Tanyscelis maculata are very similar to those of Tanyscelis maskelli. The latter can be distinguished by the much smaller eyes, the distinct marginal fringe of setae, and the longer dorsal setae on the abdomen and along the margin. Tanyscelis maculata is known from many more collections, and appears to be common in the south of Australia, whereas Tanyscelis maskelli has a more northern distribution. Populations of both species have been collected in New South Wales, although never from the same site. Except for one sample of Tanyscelis maculata collected by J. W. Beardsley, apparently from Eucalyptus macrorrhyncha (subgenus Eucalyptus: section Aromatica), and Froggatt’s (1894b) unconfirmed record from Eucalyptus gracilis (subgenus Symphyomyrtus: section Bisectae), all material of Tanyscelis maskelli and Tanyscelis maculata has been collected from eucalypt species in the section Adnataria (subgenus Symphyomyrtus).

#### Tanyscelis maskelli

(Froggatt) comb. n.

Figs 2h,i 15

Opisthoscelis maskelli Froggatt 1894b: 340–341; Froggatt 1929: 377.

##### General.

In the original description, Froggatt (1894b) states that he collected this species at Cooma, Maitland, Newcastle, and a dozen localities within a radius of 20 miles [32 km] of Sydney, but that a constant locality was Flemington on Eucalyptus siderophloia, the large-leaved box. Flemington is in western Sydney and now in the suburb of Homebush West Subsequently, Froggatt (1929) treated Flemington as the type locality, as did Miller and Gimpel (2000) in their catalogue of the Eriococcidae.

##### Gall

(Fig. 2h,i).

###### Female

(Fig. 2h). On stem. Conical (Fig. 2h) to thorn-like, height 6.4–14.1 mm, width 3.7–7.8 mm, length of basal attachment 5.2–11.0 mm. Gall opening round to oblong, 0.2–1.8 mm wide. Base of gall broad, surface rugose, distal portion attenuated and annulated, with 2–7 rings.

###### Male

(Fig. 2i). On stem and either leaf surface. Tubular, narrowing slightly towards apex, height 2.1–7.0 mm, width 1.0–2.1 mm, length of basal attachment 1.3–4.7 mm. Gall opening indistinct to round, 0.3–1.7 mm wide. Gall thin-walled and cylindrical, distal edge surrounding opening crenulate.

##### Adult female

**Figure 15.**
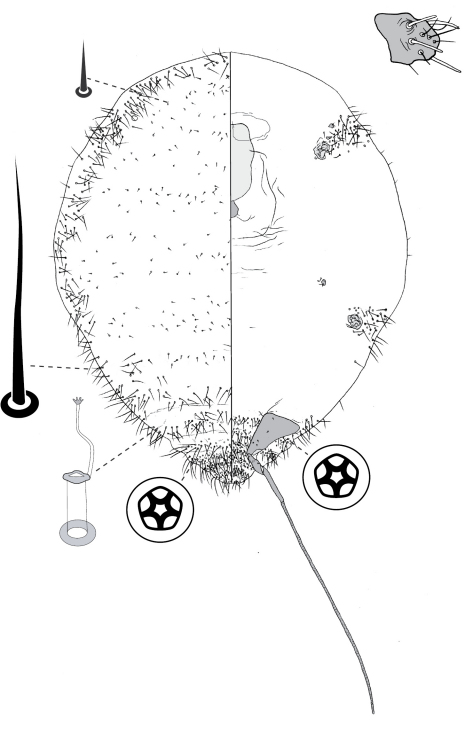
Adult female of Tanyscelis maskelli (Froggatt).

(Fig. 15) (n = 16).  Body outline ovate to turbinate, length 3.5–6.2 mm, greatest width 3.1–5.1 mm; abdomen tapered, quite small relative to thorax and head. Eyespots each 20–45 mm wide, dorsad of margin fringe setae. Antennae 1-segmented, 43–130 mm long. Frontal lobes difficult to see, each 300–350 µm long, 350–520 µm wide. Tentorial box 440–1400 mm long. Labium 125–160 mm long, 83–150 mm wide. Spiracles 120–310 mm long, 60–120 mm wide across atrium. Fore and mid legs small sclerotic protuberances, 25–55 µm long. Hind legs slender and elongate; coxa 500–1170 µm, trochanter + femur 510–1222 µm, tibia 1820–2730 µm long, tarsus 115–360 µm; translucent pores on both surfaces of tibia and tarsus; trochanter with 3 or 4 campaniform sensilla on each side; femur-tibia articulation non-functional, tibia fixed in orientation parallel to long axis of femur; claw and digitules absent. Anal opening poorly formed, 15–35 µm wide, sclerotic anal ring and anal ring setae absent.

###### Dorsum.

Derm membranous in young females, sclerotic in older specimens. Dorsal setae ranging from minute and conical to long and flagellate, 20–175 mm long; arranged in a transverse row or band across each body segment, longer setae present on abdominal segments and forming fringe along entire body margin. Macrotubular ducts 12 mm long, dermal orifice with rim ca 5 mm wide; ducts restricted to posterior abdominal segments. Microtubular ducts absent. Quinquelocular pores large, 7–11 mm in diameter, present on posterior abdominal segments.

###### Venter.

Setae flagellate, 20–165 mm long, in a transverse row across each posterior abdominal segment plus a cluster extending from each spiracle to margin, otherwise ventral body surface naked. Macrotubular ducts absent. Quinquelocular pores similar to those on dorsum, similar in distribution to ventral setae.

##### Material examined.

###### Lectotype of Opisthoscelis maskelli (here designated):

**AUSTRALIA: New South Wales:** 1 adult female (4.02 mm long, 3.24 mm wide): ex dry gall on stem, with printed label: “No. 1944 E / GALL MAKING COCCIDS. / Ophisthoscelis[sic] maskelli, Frogtt. / Male and female galls on Eucalyptus / Flemington, N.S.W.” ASCT00004851 (ASCU).

###### Paralectotypes:

**AUSTRALIA: New South Wales:** 1 adult female: same data as lectotype (ASCU); 1 adult female (slide perhaps from collection of W.M. Maskell since part of label in his handwriting): “B.M. 1945.121 / Opisthoscelis / MASKELLI / n. sp. / female / 1891 Froggatt W.M.M” and “Co Type / A gall making scale / insect, showing /the long hind / legs. / B.M. 1945.121” (BMNH); 2 adult females (slide-mounted by PJG from dry collection): ex dry stem galls with labels: “Opisthoscelis maskelli / Frogtt / on Eucalyptus siderophloia / Sydney – Australia / ex coll. W.W. Froggatt” and “Opisth Maskelli / ex Coll W. Maskell.” (BMNH).

###### Additional material:

**AUSTRALIA: New South Wales:** 3 adult females: ex galls, Eucalyptus fibrosa, 8 km N of Bargo, Hume Hwy, 9 May, 1999, LGC (ANIC); 1 adult female: ex gall, Eucalyptus fibrosa, ca 110 km N of Goulburn and 8km N of Bargo, Hume Hwy, Pheasant Nest, -34.27°; 150.65°, 10 May, 1999, LGC, Opis15 (ANIC). **Queensland:** 1 adult female: ex gall on stem, Eucalyptus sp. (ironbark), 13 km N of Injune, 7 Dec., 1993, LGC (ANIC); 15 first-instar nymphs: ex stem galls, Eucalyptus sp. (ironbark), 14 km N of Injune, 7 Dec., 1993, LGC (ANIC); 2 adult females: ex galls on petiole, Eucalyptus sp., 31 km NE of Chinchilla, Auburn Road, 6 Dec., 1993, LGC (ANIC); 3 adult females: ex stem galls, Eucalyptus sp., 34 km NNE of Chinchilla, Barakula State Forest, 5 Dec., 1993, LGC (ANIC); 1 adult female: ex pot gall with peaked cap, Eucalyptus crebra, Isla Gorge Lookout, in camping ground, 413 m, -25.19°; 149.97°, 12 Oct., 2003, LGC and M. D. Crisp, LGC00032 (ANIC); 1 adult female: ex gall, Eucalyptus melanophloia, on Sarina to Clermont Road, -21.77°; 148.79°, 2 Nov., 2003, LGC and M. D. Crisp, LGC00072 (ANIC).

##### Comments.

Adult females of Tanyscelis maskelli are very similar to those of Tanyscelis maculata [see comments under Tanyscelis maculata].

#### Tanyscelis megagibba

Hardy & Gullan sp. n.

urn:lsid:zoobank.org:act:FE152A1E-D181-48A3-8B42-007ACE65743E

Figs 3a 16 

##### Gall

(Fig. 3a).

###### Female.

On stem. A rounded swelling; gall opening slit-like; base of gall broad, gall surface striated, distal part a narrow truncate cone bearing orifice.

###### Male.

On stem; similar to gall of female but without apical cone; gall opening irregularly shaped.

##### Adult female

**Figure 16.**
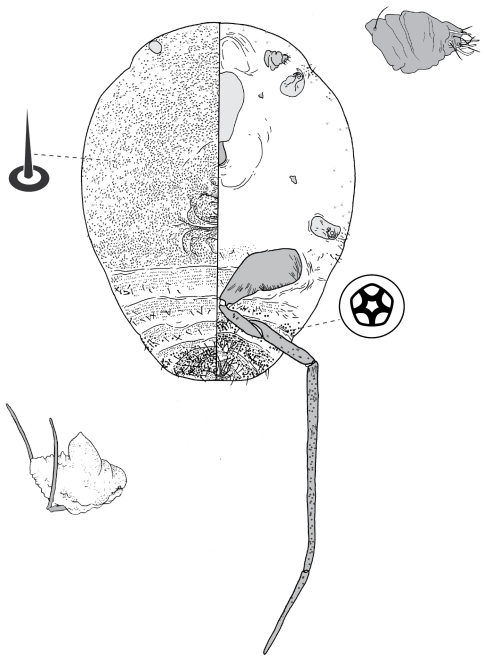
Adult female of Tansyscelis megagibba Hardy & Gullan, sp. n. Inset shows a side view of the female as it appears in life.

(Figure 16) (n = 19). Body rotund, with 3 or 4 very large dorsal humps, body margin ovate, length 1.4–3.3 mm, greatest width 0.9–ca 3 mm; abdomen appears to be tapered (truncate in all slide-preparations, but clearly tapered in photograph of adult female in life). Eyes each 60–85 µm wide, convex, with base not parallel-sided or perpedicular to body surface. Antennae 1-segmented, 95–125 mm long. Frontal lobes often difficult to detect, each ca 150 µm long, 190 µm wide. Tentorial box 325–450 mm long. Labium 115–150 mm long, 100–110 mm wide. Pump chamber 30–38 µm long, 35–40 µm wide. Spiracles 125–170 mm long, 55–100 mm wide across atrium. Fore and mid legs small sclerotic stubs, 18–45 µm long. Hind leg slender and elongate; coxa 370–430 µm long, trochanter + femur 480–550 µm long, tibia straight, 750–1000 µm long, tarsus 410–540 µm long; translucent pores scattered over both surfaces tibia, plus a few on femur and tarsus; femur-tibia articulation non-functional, base of tibia fixed at obtuse angle to femur; each side of hind trochanter with 3–6 campaniform sensilla; claw and digitules absent. Anal opening 8–20 µm wide, anal ring poorly developed, with ca 6 setae.

###### Dorsum.

Dominated by 3 or 4 large humps. Derm lightly sclerotised, densely beset with minute papillae, these diminishing in size caudad and are replaced by microtrichia on abdomen, cuticle on humps may be more heavily sclerotised than elsewhere. Dorsal setae slender, 7–53 mm long; in transverse row across each abdominal segment, scattered on head and thorax, longest setae on posterior abdominal segments. Macrotubular ducts and microtubular ducts absent. Quinquelocular pores 7–8 µm in diameter, on posterior abdominal segments.

###### Venter.

Oral lobes membranous. Setae as on dorsum, 12–80 mm long, in a transverse row across each abdominal segment, plus a few scattered along margin of head and thorax. Macrotubular ducts absent. Quinquelocular pores similar to those on dorsum, in a transverse row across each abdominal segment plus a few clustered around each spiracle, absent elsewhere.

##### Material examined.

###### Holotype (here designated):

**AUSTRALIA: South Australia:** 1 adult female (1.8 mm long, 1.2 mm wide), on slide with 2 other females, holotype closest to data label: ex gall on stem, Eucalyptus microcarpa [original label incorrectly has Eucalyptus odorata; see Comments below], Aldinga Beach, 2 Oct., 1965, H. M. Brookes, Specimen Index No. 44/65 (ANIC).

###### Paratypes:

**AUSTRALIA: South Australia:** 2 adult females, on same slide as holotype (ANIC); 6 adult females: ex stem galls, Eucalyptus microcarpa, Aldinga Beach, 9 Jan., 1964, H. M. Brookes, Specimen Index No. 4/64 (ANIC); 10 adult females: same data except 2 Oct., 1965, Specimen Index No. 44/65 (ANIC); 4 adult females: ex galls, Eucalyptus sp., Aldinga Beach, 11 Mar., 1966, HMB (ANIC); 7 adult females: ex galls, Eucalyptus sp., 1.5 km ESE of Moorlands, 6 May, 1980, PJG (ANIC).

##### Comments.

Adult females of Tanyscelis megagibba are most similar to those of Tanyscelis villosigibba, in the sense that the dorsal surface of both species is dominated by a series of large humps. These are most spectacular in mature females of Tanyscelis megagibba, but lack the dense covering of setae, each born on a raised fleshy base, that is characteristic of the adult females of Tanyscelis villosigibba. Adult females of Tanyscelis villosigibba are also distinctive in having highly convex eyes, with the base of each eye parallel-sided and perpendicular to the body surface, whereas in Tanyscelis megagibba the base of each eye is not parallel-sided. Tanyscelis megagibba is known from only two localities, ca 100 km apart, in South Australia, whereas Tanyscelis villosigibba is known from four localities in Queensland.

The original labels on the slides from two of the three collections of Tanyscelis megagibba made by HMB have the host as Eucalyptus odorata. However, her subsequent notes specify that this was a misidentification and the collections were made from Eucalyptus microcarpa (not Eucalyptus odorata).

##### Etymology.

The species name is derived from the Latin word *gibber*, meaning hump on the back. This species has impressively large dorsal humps. The name is a noun in apposition.

#### Tanyscelis mollicornuta

Hardy & Gullan sp. n.

urn:lsid:zoobank.org:act:45810F52-0775-40EE-8417-FD1D99FD9006

Fig. 17 

##### Gall.

###### Female.

On leaves. Gall a circular raised area, up to 5 mm across and 1–2 mm high; opening slit-like.

###### Male.

Not known.

##### Adult female

**Figure 17.**
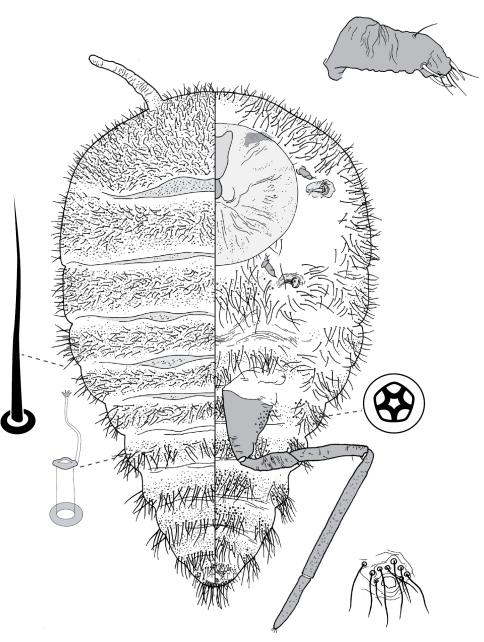
Adult female of Tanyscelis mollicornuta Hardy & Gullan, sp. n.

(Fig. 17) (n = 4). Body turbinate, margin incised at intersegmental boundaries, length 2.0–2.6 mm, greatest width 1.4–1.5 mm; abdomen tapered, about as long as head + thorax, extending beyond femur. Eyespot undetected; large (330–425 µm long), papilliform fleshy protuberance on each side of head in place of eyes. Antennae 1-segmented, 38–123 mm long. Frontal lobes difficult to see, each ca 120 µm long, 110 µm wide. Tentorial box 225–315 mm long. Labium 100–113 mm long, 60–75 mm wide. Pump chamber 28–33 µm long, 28–33 µm wide. Spiracles 85–120 mm long, 43–50 mm wide across atrium. Fore leg 50–65 µm long; mid leg 58–105 µm long. Hind leg slender and elongate; coxa conical, 300–430 µm long, trochanter + femur 580–610 µm long, tibia 630–720 µm long, tarsus 290–300 µm long; translucent pores on proximal-lateral area of each coxa, plus on both surfaces of tibia and tarsus; trochanter with 3 campaniform sensilla on each side; femur-tibia articulation functional; claw and digitules present but reduced. Anal opening ventral, 13–15 µm wide, without distinct sclerotic anal ring, surrounded by rugose area of cuticle bearing ca 10 setae.

###### Dorsum.

Derm weakly sclerotised, densely beset with small papillae. Dorsal setae flagellate, 50–165 mm long; arranged in a transverse band across each abdominal segment, densely scattered over surface of head and thorax; setae increasing in length caudad. Macrotubular ducts 15 mm long, dermal orifice with rim ca 7 mm in diameter; in transverse row across each abdominal segment, scattered over marginal and submarginal areas of head and thorax. Microtubular ducts absent. Quinquelocular pores absent.

###### Venter.

Oral lobes membranous to sclerotic, forming circular feeding pad around mouthparts. Setae 63–190 mm long, in a transverse band across each abdominal segment plus across meta- and mesothorax, and along margin of head and prothorax. Macrotubular ducts absent. Quinquelocular pores very large, ca 10 µm in diameter, on abdominal segments and medial area of mesothorax.

##### Material examined.

###### Holotype (here designated):

**AUSTRALIA: Queensland:** 1 adult female (2.0 mm long, 1.5 mm wide): ex gall on leaf, Eucalyptus populnea, 3 km WSW of Millmerran, Turallin Rd, -27.88°; 151.24°, 1 May, 1995, LGC (ANIC).

###### Paratypes:

**AUSTRALIA: Queensland:** 5 adult female: same data as holotype (ANIC).

##### Comments.

Adult females of Tanyscelis mollicornuta are most similar to those of Tanyscelis grallator [see comments under Tanyscelis grallator].

##### Etymology.

The species name is formed from the Latin word *mollis*, for soft, and *cornuta*, for horn. It refers to the pair of fleshy projections on the anterior surface of the head. The name is a noun in apposition.

#### Tanyscelis pisiformis

(Froggatt) comb. n.

Figs 3b,c 18, 19

Opisthoscelis pisiformis Froggatt 1894b: 343–344.

##### General.

When Froggatt (1894b) described Opisthoscelis pisiformis, he listed three host species of eucalypts – Eucalyptus melliodora, Eucalyptus piperita and Eucalyptus resinifera – from three different localities in New South Wales –Bathurst, Thornleigh and Sutherland. Even though we designate a lectotype below, the type locality of this species continues to encompass these three localities because the collection locality of the lectotype is not known. Although Miller and Gimpel (2000) listed the BMNH as holding syntypes, the data associated with the four BMNH collections of dry galls labelled as Opisthoscelis pisiformis either clearly show that the material was collected subsequent to description of this species or the data are inadequate to determine type status. Probably only two of the four BMNH collections belong to this species. Insects extracted from dry galls of one collection were slide-mounted by PJG in 1984 and are listed below in Material examined.

##### Gall

(Fig. 3b,c).

###### Female

(Fig. 3a). On leaf. Height 4.0–9.6 mm, width 4.0–6.4 mm, length of basal attachment 2.9–8.9 mm. Gall opening an almost closed slit in young galls, in mature galls slit-like to oblong, 0.1–1.8 mm wide, 0.6–0.8 mm long; on abaxial (lower) leaf surface. Gall opening on small raised or low conical protrusion, opposite side of leaf sub-spherical, basal attachment may be constricted; surface green, sometimes with reddish tinge, leaf glands enlarged.

###### Male

(Fig. 3b). On stem and leaf (probably either surface), height 1.1–4.0 mm, width 1.0–3.8 mm, length of basal attachment 1.4–3.9 mm. Gall conical, opening round to oblong, 0.2–1.8 mm wide.

##### Adult female

**Figure 18.**
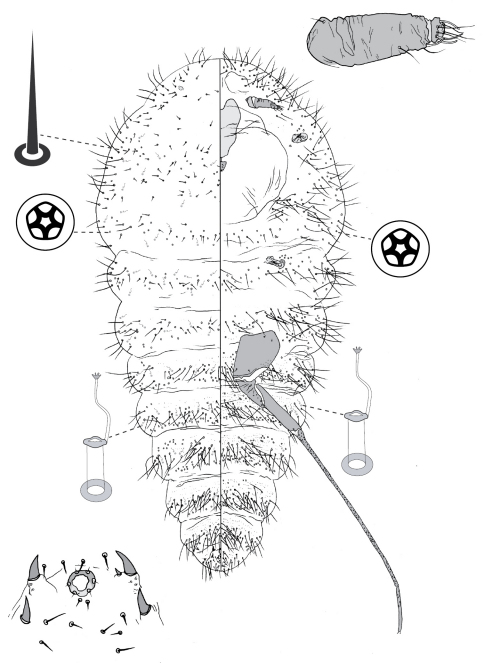
Adult female of Tanyscelis pisiformis (Froggatt).

(Fig. 18) (n = 15). Body turbinate, margin incised at intersegmental boundaries, length 2.7–3.6 mm, greatest width 1.1–2.3 mm; abdomen tapered, about as long as head + thorax, extending far beyond femur. Eyespots each 15–25 mm wide, on dorsal margin. Antennal segmentation poorly developed; each antenna 80–228 mm long. Frontal lobes difficult to see, each 125–190 µm long, 180–240 µm wide. Tentorial box 300–495 mm long. Pump chamber 34–45 µm long, 33–53 µm wide. Labium 75–160 mm long, 65–220 mm wide. Spiracles 80–125 mm long, 40–75 mm wide across atrium. Fore and mid legs small sclerotic protuberances, 12–63 µm. Hind legs slender and elongate; coxa 270–440 µm long, trochanter + femur 405–630 µm long, tibia 860–1300 µm long, tarsus 250–380 µm long; translucent pores dense throughout dorsal and ventral surfaces of tibiotarsus, a few on distolateral part of coxa; trochanter with 2 campaniform sensilla on each side; femur-tibia articulation non-functional, tibia fixed in orientation parallel to long axis of femur; claw and digitules present but reduced. Anal opening 10–18 µm wide, with anal ring, 18–32 µm wide, sclerotization of ring uneven, weaker at posteroventral end, often appearing horseshoe-shaped, anal ring with 6 minute setae. Anal area with 4 stout spines, 25–35 µm long, each at the end of a fleshy protuberance; one spine on each side of body laterad of anal ring, another on each side of body posterolateral of anal ring, each anterior anal spine with small, auxiliary spine usually present near medial edge of base plus a few minute setae on surface of protuberance.

###### Dorsum.

Derm membranous. Dorsal setae ranging from minute and conical to long and flagellate, 8–95 mm long; arranged in a transverse row or narrow band across each body segment, longer setae present on abdominal segments and along margin. Spinose seta, 5–10 µm long, found on margin of each posterior abdominal segment, absent from margin of anterior abdominal segments, head and thorax. Macrotubular ducts 15 mm long, dermal orifice with a 5 mm wide rim; in transverse row across each abdominal segment, scattered over thorax, absent from head. Microtubular ducts absent. Quinquelocular pores 5–7 µm in diameter, scattered over dorsum.

###### Venter.

Setae as on dorsum, each 12–175 mm long, in a transverse row or narrow band across each abdominal segment as well as meta- and mesothorax, along margin and submargin of head and prothorax. Macrotubular ducts similar to those on dorsum, restricted to submarginal areas of abdominal segments. Quinquelocular pores similar to those on dorsum, similar in distribution to ventral setae; pores often occurring in pairs.

##### First-instar nymph

**Figure 19.**
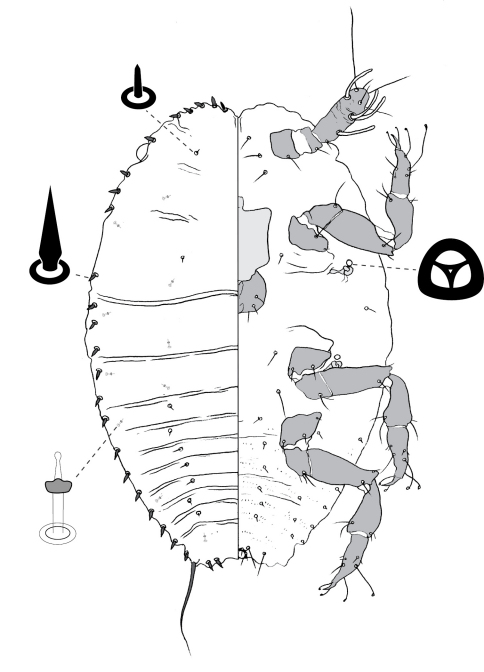
First-instar nymph of Tanyscelis pisiformis (Froggatt).

(Fig. 19) (n = 4). Body outline elliptical, anterior margin incised at midline, length 263–283 µm, greatest width 158–188 µm. Eyespots on margin, each 9–15 mm wide. Antennae 3-segmented, ca 60 mm long, with 4 fleshy setae. Tentorial box 50 mm long. Labium 20–28 mm long, 18–33 mm wide. Spiracles 11–18 mm long, 8 mm wide across atrium. Legs subequal in size: coxa 18–25 µm, with 5 setae, trochanter + femur 53–58 µm long, trochanter with 4 setae, femur with 2 setae, tibia 20–33 µm long, with 4 setae, tarsus 25–35 µm long, with 4 setae, claw 10–13 µm long; tarsal digitules capitate, unequal length, short digitule 18–20 µm long, long digitule 25–28 µm long, claw digitules capitate, each ca 15 µm long. Anal ring 9–15 µm wide, with 6 fine setae, each ca 8 µm long. Apical seta 55–65 µm long.

###### Dorsum.

Derm membranous. Dorsal setae fine and minute £1 mm long; arranged in submedial longitudinal row on each side of body, 1 seta on each side of head, prothorax, and each of abdominal segments I–VII. Microtubular ducts 4 µm long, each side of body with 1 duct on submargin of each thoracic segment plus each of abdominal segments I and V; also with 1 duct on submedial area of head, each thoracic segment and abdominal segment VIII. Marginal setae sagittate, 2–5 µm long, each side of body with ca 6 setae between midline and eyes, 4 on prothorax, 3 on mesothorax, 2 on metathorax, 1 on each of abdominal segments I–VII, and on abdominal segment VIII 1 lateral and 2 medial of apical seta, these most likely homologous to anal lobe setae.

###### Venter.

Setae hair-like, each 1–20 mm long, each side of body with 3 setae medial of scape, 1 seta medial of each coxa, 3 longitudinal rows on abdomen, each row with 1 seta on each of abdominal segments II–VII; suranal and ventral lobe setae hair-like, each ca 15 µm long. Trilocular pores 3 µm in diameter, 1 pore near each spiracle.

##### Material examined.

###### Lectotype of Opisthoscelis pisiformis (here designated):

**AUSTRALIA: New South Wales:** 1 adult female (3.5 mm long, 2.3 mm wide): ex dry gall on leaf, with printed label: “No. 1786 E / GALL MAKING COCCIDS. / Opisthoscelis pisiformis, Frogtt. / Male and female galls on Eucalyptus sp. / *N.S.W.*” ASCT00004858 (ASCU).

###### Paralectotypes:

**AUSTRALIA: New South Wales:** 2 adult female, ca 400 first-instar nymphs & embryos [10 slides], ex dry galls on leaves and stems: same data as lectotype (ASCU).

###### Additional material:

**AUSTRALIA: Australian Capital Territory:** 6 adult females: ex galls, Eucalyptus botryoides, Jervis Bay, Jervis Bay Rd, 5 Sep., 1984, PJG (ANIC). **New South Wales:** 5 adult females: ex stem galls, Eucalyptus sp., Wonboyn, 21 Feb, 1993, PJG (ANIC); 3 adult female, 25 first-instar nymphs: ex galls on leaves, Eucalyptus sp., nr Wonboyn Lake Resort, N shore, -37.24°; 149.93°, 15 Jan., 1994, PJG (ANIC); 6 adult females: ex leaf galls, Eucalyptus saligna, S. Brooman, “Strathclyde” property, bank of Clyde River, -35.52°; 150.22°, 10 Jan., 1996, PJG (ANIC); 4 adult females (on 1 slide): ex Eucalyptus robusta, Sydney, 25 Dec. 1929, WWF, #1867 (ANIC); 2 adult females (1 slide): ex Eucalyptus robusta, Sydney Harbour, WWF, #1868 (ANIC). **Locality unknown:** 5 adult females, ex dry galls with labels: “Australia / W.W. Froggatt / 95-74” and “15” (BMNH).

##### Comments.

Adult females of Tanyscelis pisiformis are very similar to those of Tanyscelis verrucula. Both species have 4 anal spines and a small but distinct anal ring that often appears horseshoe-shaped and has ca 6 setae. Both also have marginal abdominal spines that are smaller than the anal spines, and they frequently have paired quinquelocular pores. Adult females of Tanyscelis pisiformis can be distinguished from those of Tanyscelis verrucula by having (1) marginal spines restricted to the posterior abdominal segments (marginal spines also occurring along margin on head and thorax on Tanyscelis verrucula); (2) much smaller eyes (15–25 µm wide, compared to Tanyscelis verrucula with eyes 30–65 µm wide); (3) elongate flagellate setae up to 95 µm long on dorsal surface of posterior abdominal segments (shorter than 25 µm in Tanyscelis verrucula); and (4) no weakly-sclerotised pads or protuberances near the spiracles (present in Tanyscelis verrucula). Both Tanyscelis pisiformis and Tanyscelis verrucula are known from southeastern Australia, although Tanyscelis verrucula is much more widely distributed and Tanyscelis pisiformis appears to have a strictly coastal distribution and has not been collected in Victoria. The two have never been collected from the same site, although they both occur in New South Wales. Tanyscelis verrucula is known only from eucalypt species in the section Maidenaria. Most of the Tanyscelis pisiformis material was collected from unidentified hosts, but one sample was from Eucalyptus botryoides and another from Eucalyptus saligna (both in section Latoangulatae).

#### Tanyscelis spinosa

(Froggatt) comb. n.

Figs 3d,e 20

Opisthoscelis spinosa Froggatt 1894b: 341–343.
Opisthoscelis fibularis Froggatt 1894b: 344–345, syn. n.

##### General.

What we believe to be type material of Opisthoscelis spinosa does not match exactly Froggatt’s (1894b) description of the gall of the female–tapering to a sharp thorn-like apex with a minute circular orifice–because the presumed syntype galls from Eucalyptus siderophloia at Flemington have a more slit-like orifice. Assuming that Froggatt made an error in his description and that we have examined the correct type material, adult females of Opisthoscelis spinosa are indistinguishable from those of Opisthoscelis fibularis. The name Opisthoscelis spinosa has priority, as it was described on an earlier page than Opisthoscelis fibularis. Froggatt (1894b, 1921) does not compare his two species. Froggatt (1894b) appears to have confused some of his drawings of Opisthoscelis spinosa and Opisthoscelis fibularis in that the illustrated galls labelled as those of Opisthoscelis spinosa (plate XVI, fig. 10) match his description of the galls of Opisthoscelis fibularis, and the drawings of galls purported to be of Opisthoscelis fibularis (plate XVI, fig. 17) match the description for Opisthoscelis spinosa. The original description of Opisthoscelis spinosa states that this species was plentiful in several localities about Sydney and common on Eucalyptus siderophloia at Flemington, which is in western Sydney and is now in the suburb of Homebush West, whereas Opisthoscelis fibularis was described from two localities: Bathurst, New South Wales (which is about 150 km west of Flemington) and Bendigo, Victoria (Froggatt 1894b). We have examined adult female insects ex thorn-like leaf galls from localities in New South Wales, Victoria and South Australia and all specimens are similar.

##### Gall

(Fig. 3d,e).

###### Female.

On leaf. Thorn-like, height 3.1–7.3 mm, width 2.7–7.7 mm, length of basal attachment 3.4–8.1 mm, usually on adaxial (upper) leaf surface. Gall opening slit-like, ca 0.1 mm wide, 0.3–1.0 mm long. Base of gall globose and decorticated (i.e., outer tissue cracking and lifting from tissue below), distal area tapered, apex truncate.

###### Male.

On leaf (probably adaxial surface). Height 1.8–2.3 mm, width 1.6–4.5 mm, length of basal attachment 1.9–4.6 mm. Gall subconical, apex truncate, opening oblong, 0.7–1.9 mm long, 0.2–0.8 mm wide.

##### Adult female

**Figure 20.**
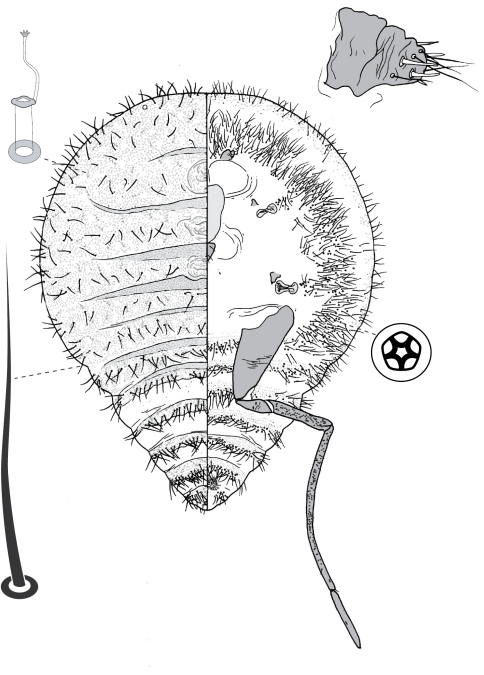
Adult female of Tanyscelis spinosa (Froggatt).

(Fig. 20) (n=28). Body turbinate, head, thorax, and anterior abdominal segments with intersegmental boundaries indistinct along margin, intersegmental boundaries incised along posterior abdominal margin; body length 1.8–3.0 mm, greatest width 1.5–2.2 mm; abdomen tapered, about as long as head + thorax. Eyespots each 20–50 µm wide, on dorsal margin. Antennae 1-segmented, 45–103 mm long. Frontal lobes difficult to see, each 165–190 µm long, 215–288 µm wide. Tentorial box 240–370 mm long. Labium 70–130 mm long, 75–163 mm wide. Pump chamber 25 µm long, 28 µm wide. Spiracles 105–188 mm long, 50–83 mm wide across atrium. Fore and mid legs each 28–106 µm long. Hind leg slender and elongate; coxa 270–666 µm long, trochanter + femur 540–980 µm long, tibia 380–1660 µm long, tarsus 320–700 µm long; translucent pores on both sides of femur and proximal proximal part of tibia; trochanter with 2 or 3 campaniform sensilla on each side; femur-tibia articulation functional; claw and digitules present but reduced. Anal opening 13–35 µm wide, without distinct sclerotic anal ring.

###### Dorsum.

Derm weakly sclerotised, densely beset with small papillae. Small medial hump present on each thoracic segment, in some specimens additional humps discernable on anterior abdominal segments. Dorsal setae flagellate, 21–202 mm long; arranged in a transverse row across each abdominal segment, scattered over surface of head and thorax; setae increasing in length caudad. Macrotubular ducts 8–11 mm long, 4–5 mm in diameter; in transverse band across each abdominal segment, scattered across head and thorax. Microtubular ducts absent. Quinquelocular pores absent.

###### Venter.

Oral lobes membranous. Setae 25–165 mm long, in a transverse row across each abdominal segment, along margin of head and thorax. Macrotubular ducts absent. Quinquelocular pores large, 8–11 µm in diameter, similar in distribution to ventral setae except absent on head, and with a cluster near each spiracle.

##### Material examined.

###### Lectotype of Opisthoscelis spinosa (here designated):

**AUSTRALIA: New South Wales:** 1 adult female (2.52 mm long, 2.06 mm wide): ex dry gall on leaf, with printed label: “No. 3093 E / GALL MAKING COCCIDS. / Opisthoscelis spinosa, Frogtt. / On the leaves of Eucalyptus siderophloia, / Flemington, N.S.W.” ASCT0004862 (ASCU).

###### Paralectotype:

**AUSTRALIA: New South Wales:** 1 adult female: same data as lectotype.

###### Lectotype of Opisthoscelis fibularis (here designated):

**AUSTRALIA: Victoria:** 1 adult female (2.95 mm long, 2.10 mm wide): ex dry gall on leaf, with printed label: “No. 1790 E / GALL MAKING COCCIDS. / Opisthoscelis fibularis, Frogtt. / Male and female galls on Eucalyptus / Bendigo, Victoria”, ASCT00004878 (ASCU)

###### Paralectotype:

**AUSTRALIA: Victoria:** 2 adult females, 6 slides with hundreds of first-instar nymphs: same data as lectotype (ASCU).

###### Additional material:

**AUSTRALIA: New South Wales:** 1 second-instar female: Eucalyptus sp. (ironbark), Arakoon Conservation Park, 27 Dec., 2005, LGC, NH117, LGC00539 (ANIC). **Queensland:** 3 adult females: Eucalyptus populifolia, Barakula, 5 Oct., 1939, No. 586 (QDPI); **South Australia:** 5 adult females: ex galls, Eucalyptus fasciculosa, Keith, 18 Feb., 1963, T. C. R. White, Specimen Index No. 15/63 (ANIC); 13 adult females, 9 second-instar females, 2 second-instar males, 55 first-instar nymphs: ex galls, Eucalyptus fasciculosa, Keith, 19 Feb., 1963, T. C. R. White, Specimen Index No. 15/63 (ANIC). **Victoria:** 7 adult females: ex galls, Eucalyptus microcarpa, 8 km W of Melton, Long Forest Road, 27 Feb., 1975, PJG (ANIC); 1 adult male: ex gall, Eucalyptus microcarpa, 8 km W of Melton, Long Forest Road, 10 Nov., 1976, PJG (ANIC); 2 adult females, 7 second-instar females: ex galls, Eucalyptus microcarpa, 8 km W of Melton, Long Forest Road, 18 Jan., 1976, PJG (ANIC); 16 adult females: ex galls, Eucalyptus microcarpa, 8 km W of Melton, near Bacchus Marsh, 6 Mar., 1976, PJG (ANIC); 1 adult female, 1 second-instar female: ex leaves, Eucalyptus ?goniocalyx (sapling), ca 20 km E of Bendigo, roadside on Calder Hwy, near Axedale Flora and Fauna Reserve, -36.78°; 144.49°, 3 Feb., 2005, PJG and NBH, NH29 (ANIC); 5 adult females: ex galls on leaves, Eucalyptus microcarpa, Long Forest, off Long Forest Road, Canopus Circuit, -37.65°; 144.50°, 12 Feb., 2005, NBH and PJG (ANIC, NMV).

##### Comments.

Adult females of Tanyscelis spinosa are most similar to those of Tanyscelis mollicornuta and Tanyscelis grallator. Adult females of Tanyscelis spinosa and Tanyscelis grallator also have the dorsum with weakly sclerotic cuticle and conical to papilliform evaginations. Adult females of Tanyscelis spinosa can be separated easily from those of Tanyscelis mollicornuta and Tanyscelis grallator by having eyes, and lacking the fleshy projections that are in the place of eyes in Tanyscelis mollicornuta and Tanyscelis grallator. Tanyscelis spinosa has been collected exclusively from eucalypt species in the section Adnataria (also true of Tanyscelis mollicornuta and Tanyscelis grallator), with the exception of a collection from a sapling that had been identified tentatively as Eucalyptus ?goniocalyx (section Maidenaria).

#### Tanyscelis tripocula

Hardy & Gullan sp. n.

urn:lsid:zoobank.org:act:E7C56FFF-20B2-43E2-B860-56FBBC928F3B

Figs 3f 21 

##### Gall

(Fig. 3f).

###### Female

On leaf. Gall circular, 3–4 mm diameter, almost level with leaf surface on orifice side, a slightly raised bump on opposite leaf surface; opening round to elongate, surrounded by raised ring of tissue, usually opening on adaxial (upper) surface of leaf. Gall tissue green but highly glaucous due to white waxy exudation on Eucalyptus cephalocarpa; gall not glaucous and becoming brown with age on Eucalyptus aromophloia.

###### Male.

On leaf, usually opening on adaxial surface. Similar to galls of females; gall surface glaucous if on Eucalyptus cephalocarpa but gall tissue red, appearing purplish due to white wax covering.

##### Adult female

**Figure 21.**
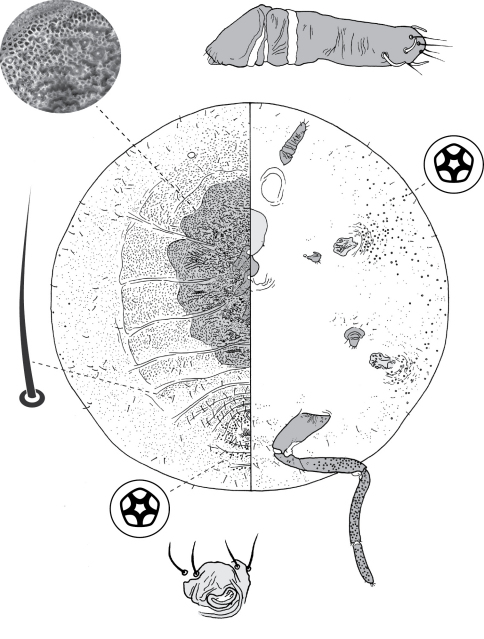
Adult female of Tanyscelis tripocula Hardy & Gullan, sp. n.

(Fig. 21) (n = 16). Body outline circular, abdomen tapered and curved dorsad in mature females, vulva and anal opening topologically dorsal, length 1.2–2.4 mm, greatest width 1.1–2.2 mm. Eyes each 28–48 µm wide, on dorsal margin. Antennal segmentation poor, each antenna appearing 3-segmented, 60–240 mm long. Frontal lobes each 170–340 µm long, 115–290 µm wide. Tentorial box 170–350 mm long. Labium 80–130 mm long, 85–120 mm wide. Pump chamber 18–23 µm long, 25–28 µm wide. Spiracles 80–175 mm long, 40–90 mm wide across atrium. Fore and mid legs small stumps, some segmentation apparent, 50–110 µm long. Hind leg with coxa 285–340 µm long, trochanter + femur 420–490 µm long, tibia slightly curved, outer margin concave, 305–410 µm long, tarsus 160–230 µm long; translucent pores dense on both surfaces of hind tarsus, tibia and all but proximal end of femur; trochanter with 2 campaniform sensilla on each side; femur-tibia articulation functional; claw and digitules present but reduced. Anal opening 8–25 µm wide, an irregular slit on dorsal body surface, surrounded by rugose sclerotic plate 40–73 µm wide; a few setae may be associated with plate, but without pores.

###### Dorsum.

With medial sclerotic shield composed of crowded nodules separated by deep fissures, with a larger deep invagination at posteromedial margin of each thoracic segment. Derm outside of shield membranous, densely beset with minute nodules. Dorsal setae flagellate, 8–50 mm long, in a transverse row across each abdominal segment, scattered over head and thorax. Macrotubular ducts absent. Microtubular ducts absent. Quinquelocular pores 7–9 µm in diameter, restricted to abdominal segment VIII and lateral areas of VII.

###### Venter.

Mature female with venter expanded relative to dorsum, so ventral body margin appears topologically dorsal. Oral lobes membranous. Setae flagellate, 8–100 mm long, a scant few on abdomen, anterior to mid legs, and along margin. Macrotubular ducts absent. Quinquelocular pores similar to those on dorsum, reasonably numerous in a band radiating from each spiracle to body margin, also a few in transverse band on each of abdominal segments V–VIII.

##### Material examined.

###### Holotype (here designated):

**AUSTRALIA: Victoria:** 1 adult female (1.35 mm long, 1.17 mm wide): ex flat, circular gall on leaf, Eucalyptus cephalocarpa, Macclesfield, Kirkpatrick’s Road, ca 300 m W of Short Road, -37.87°; 145.47°, 8 Feb., 2005, PJG and NBH, NH78 (ANIC).

###### Paratypes:

**AUSTRALIA: Victoria:** 7 adult females, 4 second-instar female, 10 adult males, 2 pupal males: same data as holotype (ANIC); 5 adult females: ex galls, Eucalyptus cephalocarpa, Macclesfield, Kirkpatrick Road, 17 July, 1976, PJG (ANIC); 7 adult females: ex galls, Eucalyptus cephalocarpa, Macclesfield, Kirkpatrick Road, 17 Oct., 1977, M. Hill (ANIC except 1 adult female BMNH, 1 adult female NMV, 1 adult female USNM); 2 adult females (1 parasitised), 3 second-instar females, 1 first-instar nymph: ex leaf gall, Eucalyptus cepahalocarpa, Cranbourne, Royal Botanic Gardens Cranbourne, -38.14°; 145.28°, 9 Feb., 2005, PJG (ANIC).

###### Additional material:

**AUSTRALIA: Victoria:** 9 adult females: ex leaf gall, Eucalyptus aromaphloia, Grampians, Victoria Valley, 400 m NW of Beehive Track, ca 1 km along Beehive Track from Serra Road, 17 Nov., 1976, PJG (ANIC); 6 adult females: ex leaf galls, Eucalyptus aromaphloia, Grampians Nat. Park, Victoria Valley, Glenelg River Road, W of Moora Moora Reservoir, -37.23°; 142.41°, 6 Feb., 2005, PJG and NBH, NH31, NH78 (ANIC).

##### Comments.

Adult females of Tanyscelis tripocula can be recognised easily by possessing a dorsal shield composed of sclerotic nodules separated by deep fissures and having a deep invagination at the posteromedial margin of each thoracic segment. Also distinctive are (1) the manner in which the posterior abdominal segments are directed dorsally in mature females (not in young adult females); (2) the shape of the anal opening, which is an irregular slit in a rugose sclerotic plate; and (3) the complete absence of tubular ducts. The phylogenetic position of Tanyscelis tripocula, as sister to all other species of Tanyscelis based on DNA data (NBH, unpublished data),is not well supported.

Galls of females on Eucalyptus cephalocarpa are smaller, flatter and glaucous compared with those on Eucalyptus aromaphloia. The adult females from galls on Eucalyptus cephalocarpa are smaller and less sclerotised than the females from Eucalyptus aromaphloia, but this may be an age effect. The type series has been restricted to specimens from Eucalyptus cephalocarpa because of the above slight differences.

Adult males were observed by PJG emerging from their galls on Eucalyptus cephalocarpa in early February 1979. They emerged head first from the gall opening, which was plugged by male’s first-instar exuviae until gall maturity. The males were capable of short jumps of 1–3 cm either forwards or backwards, as well as weak flight. Adult females also may bear their first-instar exuviae on their dorsum.

##### Etymology.

The species name is derived from the Latin word *poculum*, meaning cup and tres or *tri-* mean three. It refers to the three deep invaginations along the dorsal midline. It is a noun in apposition.

#### Tanyscelis verrucula

(Froggatt) comb. n.

Figs 3g,h 22

Opisthoscelis verrucula Froggatt 1894b: 338–339.
Opisthoscelis mammularis Froggatt 1894b: 344, syn. n.

##### General.

Here we synonymise Opisthoscelis mammularis with Opisthoscelis verrucula. Froggatt (1894b) described them as distinct species on different pages of the same article, but the two are identical based on the morphology of the adult females and their galls. Froggatt claimed that the adult female of Opisthoscelis verrucula had an “anal segment carrying an anal ring with four curved spines forming the tail.” (Froggatt 1894b: 338), whereas that of Opisthoscelis mammularis had the “tip of anal segment bearing two small reddish-brown spines curved backwards, forming a tail” (Froggatt 1894b: 344). However all specimens that we examined had one pair of large spines, each with a small spine near its base, on abdominal segment VIII, and one pair of medium-sized spines on abdominal segment VII, with the latter sometimes appearing very close to the spines on segment VIII. The type locality of Opisthoscelis verrucula is Napolean Reef, Bathurst, New South Wales, whereas that of Opisthoscelis mammularis is Bendigo, Victoria, and the types of both were collected on unidentified Eucalyptus species.

##### Gall

(Fig. 3g,h).

###### Female

(Fig. 3g). On stem, leaf petiole and midrib. Gall nipple-like, broadly rugose, shape somewhat irregular, with orifice on opposite side of leaf; height 3.8–8.7 mm, width 2.7–6.4 mm, length of basal attachment 3.2–6.7 mm. Gall orifice slit-like to oblong, 0.1–0.3 mm wide, 0.3–1.2 mm long; if gall on leaf, orifice on abaxial (lower) surface.

###### Male

(Fig. 3h). On stem and leaf (probably either surface). Height 1.5–5.0 mm, width 0.7–3.0 mm, length of basal attachment 1.1–4.2 mm. Gall cylindrical to conical, surface with shrivelled appearance, opening round to oblong, 0.1–1.3 mm wide, with opposite side of leaf swollen.

##### Adult female

**Figure 22.**
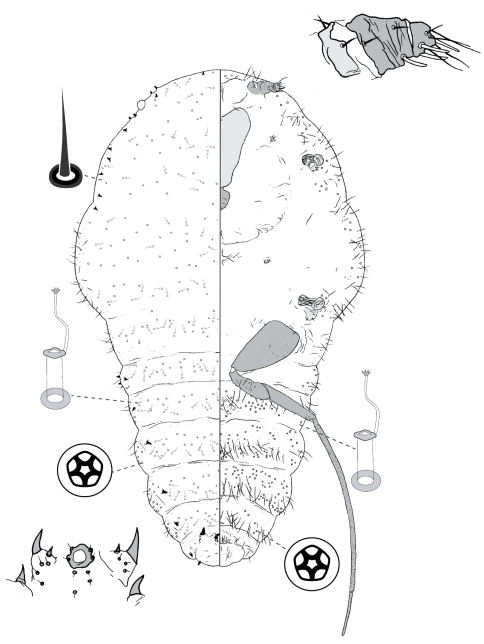
Adult female of Tanyscelis verrucula (Froggatt).

(Fig. 22) (n = 51). Body turbinate, margin incised at intersegmental boundaries, length 1.5–4.0 mm, greatest width 0.8–2.1 mm; abdomen tapered, about as long as head + thorax, extending far beyond femur. Eyespots each 30–65 mm wide, highly convex, on dorsal margin. Antennal segmentation poor, ca 3-segmented; each antenna 50–229 mm long. Frontal lobes difficult to discern, each 120–360 µm long, 110–335 µm wide. Tentorial box 230–550 mm long. Labium 50–120 mm long, 60–140 mm wide. Spiracles 75–150 mm long, 35–85 mm wide across atrium. Fore and mid legs small sclerotic protuberances, 13–80 µm wide, each with 10–18 setae. Hind legs slender and elongate; coxa 240–542 µm long, trochanter + femur 300–732 µm long, tibia 530–1540 µm long, tarsus 170–440 µm long; translucent pores scattered on both surfaces of tibia, plus a few on tarsus; trochanter with 2 campaniform sensilla on each side; femur-tibia articulation non-functional, tibia fixed in orientation almost parallel to long axis of femur; claw and digitules present but reduced. Anal opening 8–35 µm wide, with anal ring, 18–39 µm wide, sclerotisation of ring frequently uneven, weaker at posteroventral end, usually appearing horseshoe-shaped; anal ring with 4–6 minute setae. Anal area with 2 fleshy protuberances on each side of body, 1 laterad of anal ring, 1 posterolateral of anal ring, each protuberance terminating in stout spine, 20–35 µm long, each anterior protuberance with a small auxiliary spine near medial edge of base of terminal spine, plus a few minute setae.

###### Dorsum.

Derm membranous. Dorsal setae minute, 5–8 mm long; arranged in an irregular transverse row across each abdominal segment, scattered over surface of head and thorax. Stout spinose setae, 5–10 µm long, found on margin of each posterior abdominal segment plus along margin of head and prothorax. Macrotubular ducts 13–15 mm long, dermal orifice with rim 5 mm wide; in transverse row across each abdominal segment, scattered over head and thorax. Microtubular ducts absent. Quinquelocular pores 5–7 µm in diameter, scattered over dorsum.

###### Venter.

Membranous lobe-like protuberance immediately posterior of each spiracle, each protuberance having a few slender setae. Setae flagellate, 30–118 mm long, in a transverse row or narrow band across each abdominal segment, and scattered along margin of head and thorax and mesad to each metacoxa. Macrotubular ducts absent. Quinquelocular pores similar to those on dorsum, in transverse band across each of abdominal segments III–VI, a small cluster near each spiracle, a few scattered among setae along margin of head and thorax, pores often occurring in pairs.

##### Material examined.

###### Lectotype of Opisthoscelis verrucula (here designated):

**AUSTRALIA: New South Wales:** 1 adult female (3.27 mm long, 2.14 mm wide): ex dry gall on leaf, with printed label: “No. 1788 E / GALL MAKING COCCIDS. / Opisthoscelis verricula[sic], Frogtt. / Male and female galls on Eucalyptus / Bendigo / Bathurst N.S.W.” ASCT00004866 (ASCU).

###### Paralectotypes:

**AUSTRALIA: New South Wales:** 2 adult females, 1 second-instar female: same data as lectotype (ASCU); 2 adult females, numerous first-instar nymphs 5 adult males: ex leaf galls, Eucalyptus sp., Bathurst, ex WWF coll. (BMNH). The latter BMNH specimens are deemed to be paralectotypes because the collection is from the type locality and Froggatt (1894b, 1921) only ever recorded the species from this one locality.

###### Lectotype of Opisthoscelis mammularis (here designated):

**AUSTRALIA: Victoria:** 1 adult female (2.83 mm long, 2.04 mm wide): ex dry gall on leaf, with printed label: “No. 1789 E / GALL MAKING COCCIDS. / Opisthoscelis mammularis, Frogtt. / Male and female galls on Eucalyptus / Bendigo, Victoria”, ASCT00004850 (ASCU).

###### Paralectotypes:

**AUSTRALIA: Victoria:** 2 adult females, 6 slides with hundreds of first-instar nymphs: same data as lectotype (ASCU).

###### Additional material:

**AUSTRALIA: Autralian Capital Territory:** 6 adult females, 1 adult male: ex galls, Eucalyptus bridgesiana, Tidbinbilla Nature Reserve, -35.48°; 148.90°, 1 Mar., 1992, PJG (ANIC). **New South Wales:** 4 adult females, 1 second-instar female with pharate adult: ex leaf galls, Eucalyptus ?nortoni, Queanbeyan, Captains Flat Road, -35.36°; 149.27°, 7 Jan., 2001, PJG (ANIC); 4 adult females, 1 second-instar female, 45 first-instar nymphs ex stem galls, Eucalyptus sp., Tallong cementery, -34.72°; 150.08°, 16 Oct., 1993, PJG (ANIC); **Northern Territory:** 1 adult female: Eucalyptus sp. N of Alice Springs, near Todd River, 19 Nov, 1978, M. Kotzman (ANIC). **South Australia:** 18 adult females, 8 adult males: ex galls, Eucalyptus sp., Para Wirra National Park, 31 Dec., 1965, HMB, Specimen Index No. 64/65 (ANIC); 7 adult females, 2 second-instar nymphs, 2 first-instar nymphs, 2 adult males: Eucalyptus sp., Para Wirra National Park, 31 Dec., 1965 [label of several slides erroneously records date as 1 Dec., 1965], HMB, Boratynski #1743 (BMNH). **Tasmania:** 1 adult female: Eucalyptus sp., 2 km S of Dover, -43.33°; 146.99°, 1 Mar., 2004, LGC and M. D. Crisp, LGC00132 (ANIC). **Victoria:** 1 adult female: ex gall, Eucalyptus ?goniocalyx, ca 20 km E of Bendigo, near Axedale Flora and Fauna Reserve, roadside on Calder Highway, -36.78°; 144.49°, 3 Feb., 2005, PJG and NBH, NH73 (ANIC); 15 adult females: ex galls, Eucalyptus viminalis, Cranbourne, 12 Apr., 1975, PJG (ANIC); 20 first-instar nymphs: Eucalyptus viminalis, Cranbourne, 24 Nov., 1975, PJG (ANIC); 3 adult females: Eucalyptus viminalis, Cranbourne Botanic Gardens Annexe, 27 Jan., 1976, PJG (ANIC); 4 adult females: Eucalyptus viminalis, Cranbourne Botanic Gardens Annexe, 29 Aug., 1976, PJG (ANIC); 6 adult females: ex galls on leaves and stems, Eucalyptus viminalis, Cranbourne, Royal Botanic Gardens, ca 150 m SE of Trig Point Lookout, -38.13°; 145.28°, 9 Feb., 2005, PJG, NH52, NH79 (ANIC, NMV); 300 first-instar nymphs, ex gall cavity, Eucalyptus viminalis, Lang Lang, A. C. I. sandmining lease, 23 Dec., 1976, PJG (ANIC).

##### Comments.

Adult females of Tanyscelis verrucula are very similar to those of Tanyscelis pisiformis [see comments under Tanyscelis pisiformis].

There were numerous eggs in the cavity of mature galls of females of Tanyscelis verrucula collected by PJG from Eucalyptus viminalis at Cranbourne, Victoria, in November 1975. After oviposition, the body of the females shrunk to about one-fifth of their size prior to oviposition. It was estimated that a single female could lay more than 1000 eggs.

#### Tanyscelis villosigibba

Hardy & Gullan sp. n.

urn:lsid:zoobank.org:act:D95D624F-E2B4-4366-A130-60E84FBDE9B1

Figs 3i 23 

##### Gall

(Fig. 3i).

###### Female.

On stem. Globose, with truncate apex and rugose surface. Mature female almost completely fills gall cavity, positioned with three sclerotic dorsal humps positioned just below gall orifice.

###### Male.

Not known.

##### Adult female

**Figure 23.**
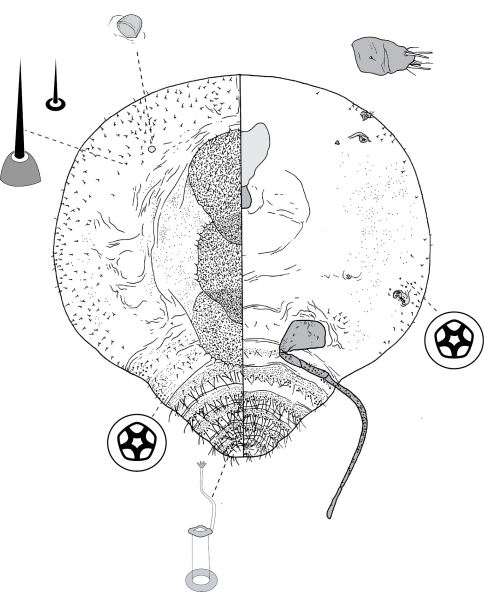
Adult female of Tanyscelis villosigibba Hardy & Gullan, sp. n.

(Fig. 23) (n = 10). Body turbinate, body margin entire, undulate (with segmental lobes) along posterior abdominal segments, length 2.2–2.9 mm, greatest width 2.0–2.6 mm; abdomen tapered, in mature females length of abdomen << length of head + thorax, abdomen extending beyond femur. Eyes highly convex, base perpendicular to body surface, parallel-sided, each 20–62 µm wide. Antennae 1-segmented, 25–85 mm long. Frontal lobes not detected. Tentorial box 375–425 mm long. Pump chamber 33–40 µm long, 33–40 µm wide. Labium 100–138 mm long, 60–93 mm wide. Spiracles 93–138 mm long, 50–93 mm wide across atrium. Fore and mid legs small sclerotic protuberances, 15–43 µm wide. Hind leg slender and elongate; coxa 395–450 µm long, trochanter + femur 340–490 µm long, tibia strongly curved, outer margin convex, 1000–1360 µm long, tarsus 220–280 µm long; translucent pores scattered on both surfaces of femur and tibia; trochanter with 2 campaniform sensilla on each side; femur-tibia articulation non-functional, base of tibia fixed; claw and digitules absent. Anal opening 8–15 µm wide, without distinct sclerotic anal ring.

###### Dorsum.

Dominated by 3 large, sclerotic and setose humps, 1 on each thoracic segment, each hump 450–910 µm long, 520–790 µm wide. Derm elsewhere membranous or weakly sclerotised. Dorsal setae robust and conical to flagellate, 10–140 mm long; flagellate setae in a transverse row across each posterior abdominal segment, robust setae mounted on small raised base, scattered over surface of head and thorax, dense on thoracic humps. Macrotubular ducts present or absent, duct 12 mm long, dermal orifice with rim 5 mm wide; if present, only 1 to a few present somewhere on abdominal segments III–VI. Microtubular ducts absent. Quinquelocular pores 7–10 µm in diameter, in a transverse row or narrow band across each posterior abdominal segment.

###### Venter.

Oral lobes membranous. Setae hair-like, each 10–130 mm long, in a transverse row across each abdominal segment; a few setae, each on a raised base, scattered along margin of head and thorax. Macrotubular ducts absent. Quinquelocular pores similar to those on dorsum, in a transverse row to narrow band across each posterior abdominal segment plus a few clustered around each posterior spiracle.

##### Material examined.

###### Holotype (here designated):

**AUSTRALIA: Queensland:** 1 adult female (2.2 mm long, 2.0 mm wide): ex gall, Eucalyptus sp. (sapling), intersection of Boomerang Road and Beenleigh-Beaudesert Road, -27.78°; 153.19°, 2 May, 1993, PJG (ANIC).

###### Paratypes:

**AUSTRALIA: Queensland:** 6 adult females, same data as holotype (ANIC); 2 adult females: Eucalyptus crebra, Barakula, 13 Apr., 1939, INSECOLL 0-067157, 0-067158 (QDPI); 5 adult females, 7 first-instar nymphs: ex stem galls, Eucalyptus sp. (ironbark), Dunmore State Forest, 2 May, 1995, G. Harper (ANIC); 1 adult female: ex gall, Eucalyptus sp., Kennedy Hwy, Hughenden–The Lynd junction, 885 m, -19.88°; 144.27°, 16 Oct., 2003, LGC and M. D. Crisp (ANIC).

##### Comments.

Adult females of Tanyscelis villosigibba are most similar to those of Tanyscelis megagibba [see comments under Tanyscelis megagibba]. Specimens of Tanyscelis villosigibba from the type locality, just south of Brisbane, lack macrotubular ducts entirely, whereas specimens from the other two localities, the closest of which is about 200 km to the west in Dunmore State Forest, have 1 or a few macrotubular ducts restricted to the dorsal surface of the abdominal segments III-VI.

##### Etymology.

The species name is a combination of the Latin words *villosus*, meaning shaggy, and *gibber*, for hump. It refers to the setose dorsal protuberances that characterise this species. The name is a noun in apposition.

## Supplementary Material

XML Treatment for Opisthoscelis

XML Treatment for Tanyscelis
